# Partial CD25 Antagonism Enables Dominance of Antigen-Inducible CD25^high^ FOXP3^+^ Regulatory T Cells As a Basis for a Regulatory T Cell-Based Adoptive Immunotherapy

**DOI:** 10.3389/fimmu.2017.01782

**Published:** 2017-12-14

**Authors:** Daniel S. Wilkinson, Debjani Ghosh, Rebecca A. Nickle, Cody D. Moorman, Mark D. Mannie

**Affiliations:** ^1^Department of Microbiology and Immunology, Brody School of Medicine, East Carolina University, Greenville, NC, United States

**Keywords:** rodent, FOXP3, regulatory T cells, experimental autoimmune encephalomyelitis/MS, tolerance, neuroimmunology

## Abstract

FOXP3^+^ regulatory T cells (Tregs) represent a promising platform for effective adoptive immunotherapy of chronic inflammatory disease, including autoimmune diseases such as multiple sclerosis. Successful Treg immunotherapy however requires new technologies to enable long-term expansion of stable, antigen-specific FOXP3^+^ Tregs in cell culture. Antigen-specific activation of naïve T cells in the presence of TGF-β elicits the initial differentiation of the FOXP3^+^ lineage, but these Treg lines lack phenotypic stability and rapidly transition to a conventional T cell (Tcon) phenotype during *in vitro* propagation. Because Tregs and Tcons differentially express CD25, we hypothesized that anti-CD25 monoclonal antibodies (mAbs) would only partially block IL-2 signaling in CD25^high^ FOXP3^+^ Tregs while completely blocking IL-2 responses of CD25^low-intermediate^ Tcons to enable preferential outgrowth of Tregs during *in vitro* propagation. Indeed, murine TGF-β-induced MOG-specific Treg lines from 2D2 transgenic mice that were maintained in IL-2 with the anti-CD25 PC61 mAb rapidly acquired and indefinitely maintained a FOXP3^high^ phenotype during long-term *in vitro* propagation (>90% FOXP3^+^ Tregs), whereas parallel cultures lacking PC61 rapidly lost FOXP3. These results pertained to TGF-β-inducible “iTregs” because Tregs from 2D2-FIG *Rag1^−^*^/^*^−^* mice, which lack thymic or natural Tregs, were stabilized by continuous culture in IL-2 and PC61. MOG-specific and polyclonal Tregs upregulated the Treg-associated markers Neuropilin-1 (NRP1) and Helios (IKZF2). Just as PC61 stabilized FOXP3^+^ Tregs during expansion in IL-2, TGF-β fully stabilized FOXP3^+^ Tregs during cellular activation in the presence of dendritic cells and antigen/mitogen. Adoptive transfer of blastogenic CD25^high^ FOXP3^+^ Tregs from MOG35-55-specific 2D2 TCR transgenic mice suppressed experimental autoimmune encephalomyelitis in pretreatment and therapeutic protocols. In conclusion, low IL-2 concentrations coupled with high PC61 concentrations constrained IL-2 signaling to a low-intensity range that enabled dominant stable outgrowth of suppressive CD25^high^ FOXP3^+^ Tregs. The ability to indefinitely expand stable Treg lines will provide insight into FOXP3^+^ Treg physiology and will be foundational for Treg-based immunotherapy.

## Introduction

CD4^+^ CD25^+^ FOXP3^+^ regulatory T cells (Tregs) mediate an integral role in controlling autoimmunity and chronic inflammatory disorders (1, 2). The importance of Tregs is illustrated by loss-of-function mutations in *Foxp3* that cause early-onset, fatal, multi-organ autoimmune disorders IPEX (immunodysregulation polyendocrinopathy enteropathy X-linked syndrome) in humans and scurfy in mice (3). Moreover, dysfunctional Treg responses have been implicated in susceptibility to several autoimmune diseases including multiple sclerosis and type 1 diabetes (4). Treg-mediated suppressive activity has promise for translation as an immunotherapy for autoimmune disease and other chronic inflammatory disorders. Treg adoptive immunotherapy is based on the concept that Tregs can be isolated or induced *ex vivo*, expanded as a stable subset *in vitro*, and then infused into a syngeneic recipient to inhibit autoimmune inflammation. Treg immunotherapy has shown encouraging results in preclinical models of autoimmune disease such as experimental autoimmune encephalomyelitis (EAE), type 1 diabetes, and colitis (5–7). In early phase clinical trials, Treg-based immunotherapies for transplantation and type 1 diabetes have had favorable outcomes (8, 9). However, adoptive Treg immunotherapy has yet to be approved as a treatment option due to technical limitations associated with Treg instability during *in vitro* expansion.

FOXP3 is expressed in a canonical lineage of suppressive Tregs and is an obligate requirement for adaptive self-tolerance. However, FOXP3^+^ Tregs exhibit phenotypic and functional plasticity (10, 11), which represents a primary obstacle for development of Treg-based immunotherapy. *In vivo* fate-mapping studies that tracked FOXP3^+^ Tregs showed that strong cellular activation in pro-inflammatory environments caused the loss of the immunosuppressive FOXP3 phenotype, such that ex-Tregs downregulated FOXP3 expression and acquired effector function (12). Indeed, Treg lines lost FOXP3 expression when cultured in IL-2, especially when undergoing multiple *in vitro* activations (13). The concern is that conversion of FOXP3^+^ Tregs to effector “ex-Tregs” may exacerbate autoimmune disease. Instability of Treg lineages may reflect intrinsic loss of the FOXP3^+^ Treg phenotype on a per cell basis. However, instability of continuous Treg lines may also reflect overgrowth of stable Tregs by effector T cells because Tregs exhibit proliferative anergy, whereas conventional T cell (Tcon) subsets exhibit hyper-proliferative growth rates. Various therapeutic strategies have attempted to directly manipulate Treg stability by administration of low-dose IL-2 or IL-2/anti-IL-2 immune complexes to limit IL-2 availability and favor Treg responses in animal models and in the clinic (14–17). Additional studies revealed that the immunosuppressive drug rapamycin may favor predominance of Tregs over Tcon subsets (18). However, these strategies are not sufficient to derive Treg cultures suitable for adoptive immunotherapy.

Two distinct lineages of Tregs are defined based upon the site of initial differentiation (19). Thymically derived Tregs (tTregs) differentiate in the thymus whereas induced Tregs (iTregs) arise *de novo* in extrathymic tissues or are induced *in vitro*. The tTreg subset is advantageous because tTregs are thought to be more stable than iTregs (20, 21). Expansion of antigen-specific tTregs nonetheless is technically challenging because, like iTregs, tTregs gradually downregulate FOXP3 expression during cellular activation and *in vitro* expansion (13). The challenge is the derivation of antigen-specific lines of either Treg lineage, because antigen-specific Tregs are more suppressive than non-specific polyclonal Tregs in antigen-bearing tissues (6, 22). Indeed, the use of iTregs, inducible by antigen, may provide advantages for derivation of antigen-specific Tregs. The hurdle is to achieve stability of TGF-β-iTregs during long-term *in vitro* culture so that one can exponentially expand rare antigen-specific clonotypes to achieve antigen-specific, stable FOXP3^+^ Treg lines. Derivation of antigen-specific Tregs will require long-term clonotypic expansion *in vitro*, due to the limited frequencies of antigen-specific precursors. The application of Tregs in adoptive immunotherapy for MS for example would hinge on the ability to stabilize myelin-specific iTregs during sustained cycles of propagation and reactivation, which is the main focus of this study.

IL-2 is necessary for maintenance of both Tregs and many Tcon subsets *in vitro*. Compared to other T cell lineages, Tregs have superior sensitivity to limited IL-2 concentrations because many FOXP3^+^ Tregs constitutively express superlative levels of the IL-2 receptor α chain CD25. Although some quasi-stable FOXP3^+^ T cells lack robust levels of CD25, the CD25^high^ FOXP3^+^ phenotype represents a prototypic phenotypic marker for immunosuppressive Tregs. Although CD25 is also transiently expressed on activated effector T cell lineages, CD25 is rapidly down-regulated on Tcons during reversion to a quiescent resting state. The CD25^high^ phenotype reflects a major immunoregulatory strategy because superlative CD25 expression enables Tregs to monopolize and consume the local IL-2 supply to ensure regional Treg dominance coupled with IL-2 starvation and death of Tcon subsets (23). In this regard, the IL-2/CD25 pathway may represent the critical regulatory nexus controlling the differential growth and survival of Treg and Tcon subsets. However, we currently lack an adequate understanding of how the IL-2/CD25 pathway controls the relative dynamics of Treg or Tcon growth.

Differential expression of CD25 and the consequent differences in IL-2 potency may provide a means to directly manipulate the balance of Treg and Tcon subsets in cell culture. This study is based on the hypothesis that CD25^high^ FOXP3^+^ Tregs will exhibit relatively high degrees of resistance to CD25 blockade whereas CD25^intermediate^ Tcon subsets will be fully susceptible to CD25 blockade and will selectively die due to cytokine starvation. In support of this hypothesis, the anti-mouse CD25 monoclonal antibody (mAb) PC61 supported the differential expansion of FOXP3^+^ T cells and was the key agent necessary to generate long-term, FOXP3^+^ Treg lines that were stable over several months of *in vitro* propagation. At high PC61 concentrations and low IL-2 concentrations, IL-2-dependent Treg proliferation was dominant over Tcon proliferation, and these differential growth rates enabled Tregs to progressively dominate mixed cultures. Continuously propagated Treg lines progressively expressed Treg-associated markers Neuropilin-1 (NRP1) and Helios (IKZF2). Treg lines were reactivated in the presence of TGF-β and expanded *in vitro* in the presence of PC61 without showing any decrease in FOXP3 expression as a percentage of the T cell population or on a per cell basis. Tregs propagated in culture for extended periods of time retained suppressive capabilities as shown by *in vitro* suppression assays and by adoptive transfer of suppression in the C57BL/6 model of EAE.

## Materials and Methods

### Mice

C57BL/6 mice, MOG35-55 specific TCR transgenic 2D2 mice [B6-Tg(Tcra2D2, Tcrb2D2)1Kuch/J] (Stock Number 006912), OVA323-339 specific TCR transgenic OT-II mice (Stock Number 004194), B6.129S7-*Rag1^tm1Mom^*/J (Stock Number 002216), and Foxp3-IRES-GFP knock-in (FIG) mice (B6.Cg-*Foxp3*^tm2Tch^/J, Stock Number 006772) were obtained from Jackson Laboratory (Bar Harbor, ME, USA) and were maintained as a colony in the Department of Comparative Medicine. In the 2D2-FIG and OTII-FIG strains, the FIG GFP reporter was used as a surrogate marker of FOXP3 expression. Routine screening of 2D2 mice was performed by FACS analysis of PBMC by use of antibodies specific for TCR Vβ11 and/or Vα3.2. Routine screening of OT-II mice was performed by FACS analysis of PBMC by use of antibodies specific for TCR Vβ5.1/5.2 and Vα2. The FIG genotype was screened by use of forward (CAC CTA TGC CAC CCT TAT CC) and reverse (ATT GTG GGT CAA GGG GAA G) primers. Animal care and use was performed in accordance with approved animal use protocols and guidelines of the East Carolina University Institutional Animal Care and Use Committee.

### MOG35-55, OVA323-339, TGF-β, and IL-2

Synthetic MOG35-55 (MEVGWYRSPFSRVVHLYRNGK) and OVA323-339 (ISQAVHAAHAEINEAGR) peptides were obtained from Genscript (Piscataway, NJ, USA). Recombinant rat TGF-β1 was expressed by use of transfected human embryonic kidney (HEK) cells. TGF-β1 was expressed as described (24, 25). This expression vector encoded a rat serum albumin leader sequence, an 8-histidine purification tag, the latency-associated peptide (LAP), the native RHRR cleavage site, and the C-terminal TGF-β1 sequence. A C32S substitution in the LAP domain enabled high level expression. The protein was expressed in HEK supernatants, purified on Ni-NTA affinity columns, and was activated by 10 min of exposure to 70°C. The bioactivity of each TGF-β1 preparation was verified by induction of FOXP3 in cultures of MOG-stimulated 2D2-FIG splenocytes (SPL). Recombinant rat IL-2 (~10–30 U/ml) was derived from a baculovirus expression system and was used routinely in bulk T cell culture (26). Recombinant murine IL-2 was purified from a stable transfected HEK293F cell line.

### Generation, Purification, and Administration of mAbs and PC61scFv

The PC61-5.3 anti-CD25 rat IgG1(λ) hybridoma (27), the 7D4 anti-CD25 rat IgM(κ) hybridoma, and the 1D11.16.8 anti-mouse-TGF-β1/2/3 mouse IgG1 hybridoma (28, 29) were obtained from ATCC. The 3C7 anti-CD25 rat IgG2b(κ) hybridoma was a generous gift from Dr. Ethan Shevach (NIH). All hybridomas were subcloned twice to ensure stability. Hybridoma supernatants were clarified at 7,200 × *g*, precipitated with 50% ammonium sulfate, and dissolved in PBS. mAbs were purified on protein G agarose columns, eluted with 200 mM glycine at pH 3.0, and neutralized by 1 M Tris buffer of pH 9.0. The purity of these mAb was verified by SDS-PAGE. Specific activities of PC61 preparations were measured by staining of murine CD25^+^ T cells with serial 1/2 log dilutions of the mAb. After washing, PC61-stained T cells were labeled with a PE-conjugated goat anti-rat IgG (H + L) secondary antibodies followed by flow cytometric analysis.

The PC61scFv gene encoded (from N-terminus to C-terminus) the rat serum albumin signal peptide, a polyhistidine affinity purification tag, the PC61 variable light chain domain, a (Glycine_4_Serine_1_)_4_ linker, and the PC61 variable heavy chain domain. The PC61 VL and VH domain sequences were described previously (30). The sequence was as follows: M-A-K-W-V-T-F-L-L-L-L-F-I-S-G-S-A-F-S-H-H-H-H-H-H-H-H-H-(variable light chain domain)-G-G-G-G-S-G-G-G-G-S-G-G-G-G-S-G-G-G-G-S-(variable heavy chain domain)-A-K-G-G-G-S-E-G-G-G-S-E-G-G-G-S-G. The PC61scFv gene sequence was cloned into the pIRES AcGFP1 expression vector (Clontech) and used to stably transfect HEK293F cells. PC61scFv was purified using a column loaded with Ni-NTA resin, and purity was measured using SDS-PAGE. PC61scFv specificity and activity was validated by inhibition of IL-2-dependent proliferation of an IL-2-dependent cell line.

### Flow Cytometric Analyses of SPL and PBMC

Cells were washed in HBSS with 2% heat-inactivated FBS and stained for 1 h at 4°C in the dark with designated cocktails of fluorochrome-conjugated antibodies, including those specific for Vβ11 (KT11), Vβ5.1 (MR9-4), CD25 (PC61), CD25 (3C7), LAP (TW7-16B4), GARP (F011-5), GITR (DTA-1), PD-1 (29F-1A12), Nrp1 (3E12), Helios (22F6), CD69 (H1.2F3), ICOS (C398.4A), CTLA4 (UC10-4B9), TIGIT (1G9), CD44 (IM7), CD62L (MEL-14), CD45.1 (A20), and goat anti-rat IgG. Cells were then washed three times with HBSS/2% FBS. Data were collected by use of a Becton-Dickinson LSRII flow cytometer (San Jose, CA, USA) and analyzed by use of FlowJo software.

### Generation and Maintenance of Treg Lines

Naïve SPL were harvested from 2D2-FIG mice, 2D2-FIG-*Rag1^−^*^/^*^−^* mice, OTII-FIG mice, or FIG mice. These SPL were activated at a density of 2 × 10^6^/ml in complete RPMI (cRPMI; 10% heat-inactivated fetal bovine serum, 2 mM glutamine, 100 µg/ml streptomycin, 100 U/ml penicillin, 50 µM 2-ME) for 3–4 days with 1 µM MOG35-55, 100 nM OVA323-339, or 2.5 µg/ml Con-A, as indicated. Naïve FOXP3^null^ T cells were isolated from 2D2-FIG SPL by FACS to support the *in vitro* generation of TGF-β-induced iTregs and thereby exclude the contribution of thymic/natural tTregs/nTregs in designated experiments. After the initial activation, T cells were passaged every 3–4 days in rat IL-2 and were periodically reactivated every 2–4 weeks by reactivation with specific antigen or mitogen in a 3- to 4-day culture with irradiated splenic APC to drive cellular activation and expand T cell numbers. The initial activation also included 10 nM TGF-β to elicit Treg differentiation, but TGF-β was not added during the subsequent maintenance passages of the line in rat IL-2. PC61 or a designated anti-CD25 antibody (10 µg/ml; 65 nM) was included in the activation and/or maintenance cultures as designated. Cells were propagated every 3–4 days in maintenance cultures containing cRPMI and rat IL-2 along with 10 µg/ml PC61 (or designated anti-CD25 antibody). For polyclonal Treg lines derived from FIG mice, CD4^+^ cells were purified 10 days post-activation using magnetic bead positive selection (Miltenyi Biotec). Unless otherwise noted, subsequent reactivation of Tregs consisted of co-culturing irradiated SPL (2 × 10^6^/ml) and 2D2-FIG Tregs (2 × 10^5^/ml) in the presence of 1 µM MOG35-55, TGF-β (as designated), and rat IL-2 with or without PC61 as designated. After 3 days, activated Tregs were passaged into cRPMI containing rat IL-2 and 10 µg/ml PC61.

### *In Vitro* Suppression Assay

CD45.2 2D2-FIG Tregs were cultured in PC61 and IL-2 for either 13 or 40 days, and a control line of CD45.2 2D2-FIG Tcons were cultured in IL-2 for 13 days. These Treg and control lines were used to test the ability of Tregs to suppress naïve 2D2-FIG T cell activation. CTV-stained CD45.1 2D2-FIG SPL (150,000/well) were used as responders. CTV-labeled responders were cultured with CD45.2 2D2-FIG Tregs or with CD45.2 2D2-FIG Tcons (25,000/well) in the presence or absence of 1 µM MOG35-55 and rat IL-2. After 5 days of culture, proliferation of CD45.1 2D2 T cells was analyzed by measuring CTV dilution.

### Induction and Assessment of EAE

CFA (Incomplete Freund’s Adjuvant plus 4 mg/ml heat-killed *Mycobacterium tuberculosis* H37Ra, BD Biosciences, Franklin Lakes, NJ, USA) was mixed 1:1 with MOG35-55 in phosphate-buffered saline. The CFA/antigen mixture was emulsified by sonication. EAE was elicited in B6 mice by injection of 100 or 200 µg MOG35-55 in a total volume of 100 µl emulsion *via* three subcutaneous injections of 33 µl across the upper back. Each mouse received separate injections (200 ng i.p.) of *Pertussis toxin* on days 0 and 2. All immunizations were performed under isoflurane anesthesia (Abbott Laboratories, Chicago, IL, USA). Mice were assessed daily for clinical score and body weight. The following scale was used to score the clinical signs of EAE: 0, no disease; 0.5, partial paralysis of tail without ataxia; 1.0, flaccid paralysis of tail or ataxia but not both; 2.0, flaccid paralysis of tail with ataxia or impaired righting reflex; 3.0, partial hind limb paralysis marked by inability to walk upright but with ambulatory rhythm in both legs; 3.5, same as above but with full paralysis of one leg; 4.0, full hindlimb paralysis; 5.0, total hindlimb paralysis with forelimb involvement or moribund. A score of 5.0 was a humane endpoint for euthanasia.

The incidence of EAE reflected the number of mice afflicted with EAE compared to the total group size. Cumulative EAE scores were calculated by summing daily scores for each mouse across the time course of disease. Maximal scores were calculated as the most severe EAE score for each mouse. Mice that did not exhibit EAE had a score of 0 for the cumulative and maximal scores, and these scores were included in the group average. Mice that exhibited humane endpoints as assessed by body weight loss, body score, or clinical score of 5.0 were subjected to humane euthanasia and were omitted from scoring thereafter. Time-course graphs portrayed daily mean maximal scores. To calculate percent maximal weight loss, 100% body weight was assigned as the maximal body weight obtained from day 1 through day 10, and daily body weights were calculated for each day after normalization to this 100% value. The minimum body weight was defined as the lowest body weight after normalization to the 100% value during the span of day 11 until the end of the experiment. Maximal weight loss was calculated by subtraction of the normalized minimum value from the 100% value. Average weight loss was calculated as the average of daily body weight measurements from day 11 until the end of the experiment, subtracted from the 100% maximal body weight.

### Statistical Analysis

To determine statistical significance, comparisons among three or more groups were analyzed by use of ANOVA, and comparisons between two groups were analyzed by a Student’s *t*-test. A *p*-value <0.05 was considered significant. For EAE mean clinical scores and percent initial body weight, error bars represent SEM. For all other data, error bars represent SD.

## Results

### The Concept of a Treg Window

**Figure 1:**
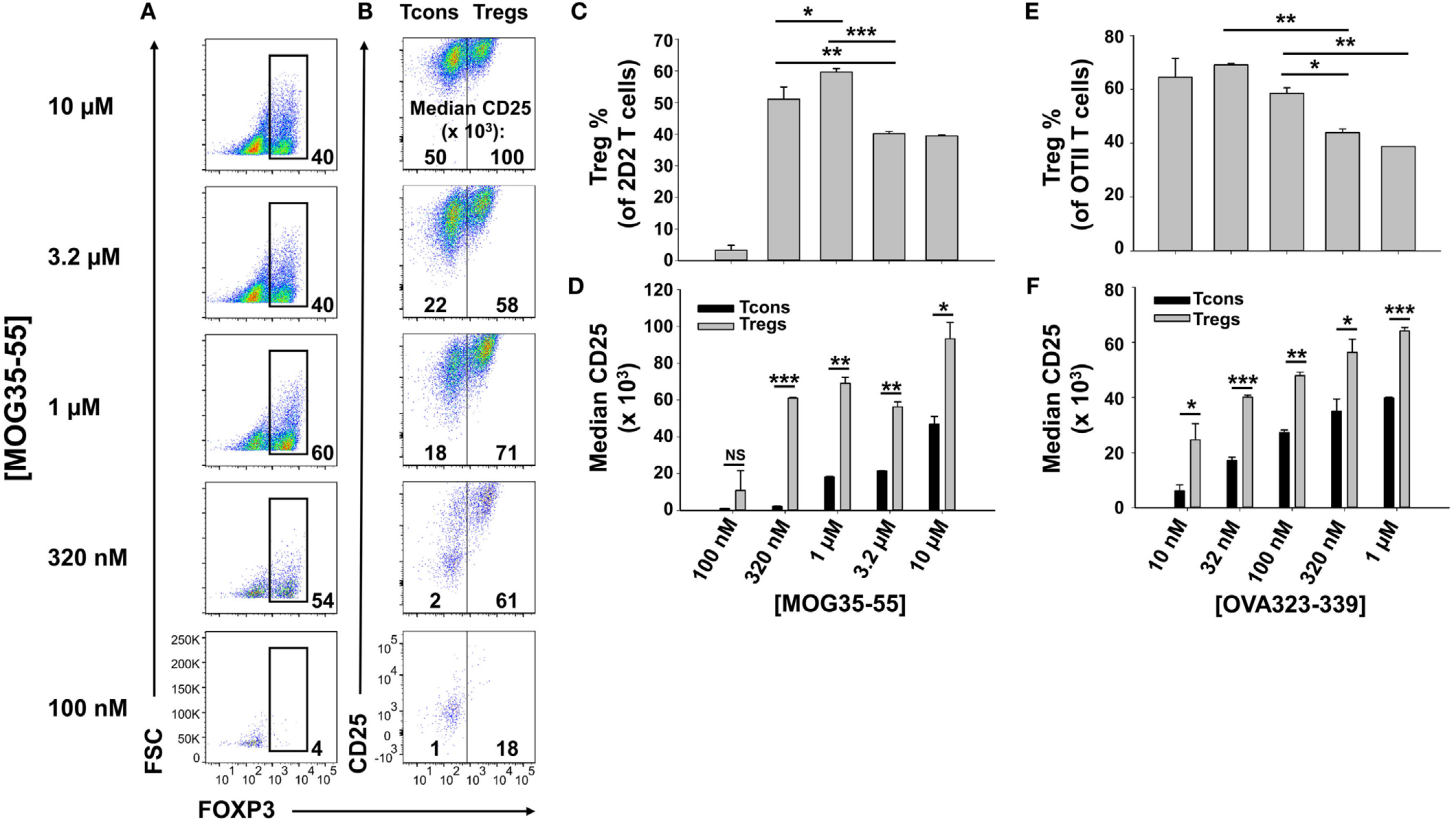
Intermediate concentrations of antigen induced higher percentages of FOXP3^+^ regulatory T cells (Tregs). 2D2-FIG **(A–D)** or OTII-FIG **(E,F)** splenocytes were activated at a density of 2 × 10^6^ cells/ml cRPMI in the presence of 10 nM TGF-β with the indicated concentration of MOG35-55 or OVA323-339. After 3 days, Vβ11^+^ (2D2) or Vβ5.1^+^ (OT-II) T cells were analyzed for expression of FOXP3 **(A,C,E)** and CD25 **(B,D,F)**. Numbers at the bottom of each dotplot in panel **(A)** represent the percentages of FOXP3^+^ T cells, and the numbers in panel **(B)** represent the median fluorescence intensity of CD25 expression within the FOXP3^−^ [conventional T cell (Tcon)] and FOXP3^+^ (Treg) populations. *p < 0.05, **p < 0.01, ***p < 0.001. These data are representative of three independent experiments.

To optimize antigen-dependent induction of Tregs, designated concentrations of MOG35-55 were used to activate 2D2-FIG SPL in the presence of 10 nM TGF-β (Figure 1). 2D2-FIG T cells are specific for MOG35-55 and have an IRES-GFP reporter knock-in immediately downstream of the FOXP3 gene. The CD4^+^ T cell compartment of 2D2-FIG mice typically contained ≤1% FOXP3^+^ T cells at steady state. After 3 days, 320 nM or 1 µM MOG35-55 elicited percentages of FOXP3^+^ Tregs (54 and 60%, respectively) that were significantly higher than the 40% Tregs induced by the higher concentrations of 3.2 and 10 µM MOG35-55 (Figures 1A,C). Thus, the antigen-dependent induction of FOXP3 resembled a bell-shaped curve. MOG-induced FOXP3 expression was entirely dependent upon TGF-β (25). The antigen-dependent expression of CD25 was at least twofold higher on Tregs than on Tcons, and this difference was maximal at intermediate antigen concentrations of MOG35-55 (320 nM–3.2 µM) (Figures 1B,D). Similar patterns were noted regarding the concentration dependence of OVA323-339 for CD25 expression on OVA-specific OTII-FIG T cells in the presence of TGF-β (Figures 1E,F). A lower range of OVA323-339 concentrations was optimal because OT-II T cells have more potent antigenic responsiveness than 2D2 T cells. These data confirm the prediction that activated 2D2 and OT-II Tregs exhibit superior CD25 expression compared to Tcon subsets.

To realize the goal of achieving long-term stable growth of FOXP3^+^ Tregs, we reasoned that an anti-CD25 mAb would differentially block the IL-2-dependent growth and survival of Treg and Tcon subsets. High levels of CD25 were postulated to confer partial resistance to anti-CD25 mAbs among Tregs while completely blocking IL-2-dependent responses of Tcons. Based on this concept, we predicted that a “Treg window” could be operationally defined by adjusting the relative concentrations of IL-2 and anti-CD25 mAb that would favor dominant survival and expansion of Tregs.

### The Anti-CD25 PC61 mAb Stabilized Short-term Treg Cultures

**Figure 2:**
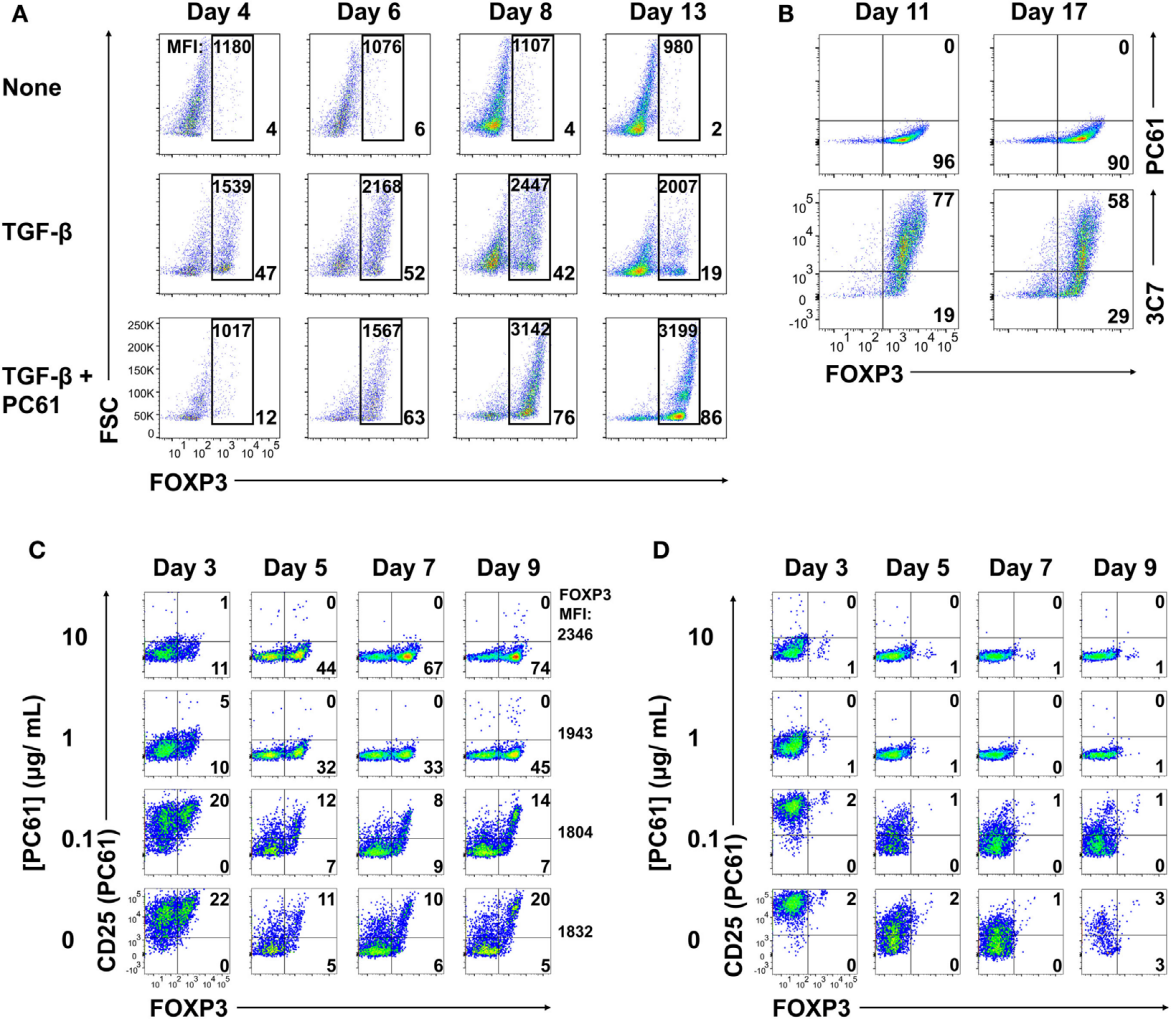
The anti-CD25 PC61 monoclonal antibody (mAb) selectively favored regulatory T cell (Treg) dominance in mixed Treg/conventional T cell cultures. **(A–D)** 2D2-FIG splenocytes were activated at a density of 2 × 10^6^ cells/ml in cRPMI with MOG35-55 (1 µM) in the presence or absence of TGF-β (10 nM) and/or designated concentrations of PC61 (10 µg/ml; 65 nM, unless designated otherwise). Antigen and TGF-β were present only in the first activation culture and not thereafter. After 3–4 days of activation, cells were passaged at a density of 10^6^ cells/ml in media containing rat IL-2 with or without PC61. Cultures initiated with PC61 were continued with PC61 during passage into the same conditions every 2–4 days. **(A)** Cells were analyzed on days 4, 6, 8, and 13 post-activation for FOXP3 expression by gating on Vβ11^+^ cells. The percentage of FOXP3^+^ Tregs compared to total Vβ11^+^ cells is indicated in the bottom right of each dotplot. The geometric mean fluorescence intensity of GFP expression is indicated at the top of each gate. **(B)** The initial T cell activation culture did not include PC61, and T cells were then passaged every 3–4 days in rat IL-2 and 10 µg/ml PC61. Vβ11-gated T cells were analyzed on day 11 and 17 for expression of CD25 by PE-conjugated 3C7 or APC-conjugated PC61 anti-CD25 mAb. **(C,D)** The initial activation culture with **(C)** or without **(D)** TGF-β included concentrations of PC61 (10, 1, 0.1 µg/ml, or none) that were maintained throughout the course of the experiment. On days 3, 5, 7, and 9, Vβ11^+^ cells were analyzed for FOXP3 expression and for binding of an APC-conjugated anti-CD25 PC61 mAb. These data are representative of three independent experiments.

To test whether an anti-CD25 antibody could be used to enrich Treg lines, 2D2-FIG SPL were activated with MOG35-55 (Figure 2A, top row), MOG35-55 and TGF-β (second row), or MOG35-55, TGF-β, and the anti-CD25 antibody PC61 (third row) for 3 days and then passaged into IL-2 containing media (without antigen or TGF-β) in the absence (rows 1 and 2) or presence (row 3) of PC61. In cultures of TGF-β-induced FOXP3^+^ Tregs (second row), Treg percentages gradually waned during subsequent propagation in IL-2 from a frequency of 47% on day 4 to 19% by day 13. Treg percentage values are given in the bottom right of each panel. In TGF-β-induced cultures that were continuously supplemented with 10 µg/ml (65 nM) of PC61 (bottom row), Treg differentiation was inhibited by PC61 during the initial 3-day activation culture. However, during the subsequent culture with PC61 and IL-2, the Treg frequency increased from 12% on day 4 to 86% by day 13. The elevated Treg frequencies were associated with stronger GFP^bright^ fluorescence which is a correlate of FOXP3 expression (values given at top of each panel). These data indicated that PC61 enabled the selective enrichment of Tregs during propagation in IL-2 over 13 days of culture. Cultures seeded in the absence of TGF-β were used as a reference to gauge the GFP^+^ window. These data revealed that CD25 was important for the initial induction of Tregs, because PC61 inhibited FOXP3 differentiation in the initial 3-day activation. Conversely, partial blockade of the IL-2/CD25 signaling pathway had the opposite activity in the IL-2 propagation cultures, because PC61 was needed to maintain FOXP3^+^ Tregs during culture in IL-2. Overall, these data showed that PC61, when used to supplement IL-2 expansion cultures, selected FOXP3^+^ Tregs as the dominant lineage.

PC61 lacked cytotoxic activity *in vitro*, although whether PC61 neutralized or down-modulated CD25 was uncertain. To assess this issue, 2D2-FIG SPL were activated for 3 days with 1 µM MOG35-55 and 10 nM TGF-β and then were passaged every 3–4 days in IL-2 and 10 µg/ml PC61 (Figure 2B). Again, antigen and TGF-β were not included in the IL-2 expansion phase of these experiments. Analyses on days 11 and 17 revealed that the line was almost entirely comprised of FOXP3^+^ Tregs (>90%). A PE-conjugated 3C7 anti-CD25 mAb, which recognizes a site on CD25 distinct from the PC61 epitope, revealed high levels of CD25 on these Tregs despite the continuous culture in PC61 and the saturation of CD25 with PC61 (Figure 2B, bottom panels). Conversely, an APC-conjugated PC61 did not bind CD25 because PC61-specific epitopes on CD25 were saturated with unlabeled PC61 mAb that had bound CD25 during the culture phase (Figure 2B, top panels). These data indicate that PC61 facilitates Treg selection, at least in part, by functional neutralization of CD25 *in vitro*.

An important question was whether Treg enrichment required PC61-mediated saturation of CD25 (Figure 2C). To assess this question, 2D2-FIG T cells were activated for 3 days with MOG35-55 and TGF-β in the presence or absence of designated PC61 concentrations. As noted before, high concentrations of PC61 inhibited Treg induction when assessed on day 3. The T cells were then passaged into IL-2 on day 3 with the same PC61 concentrations but without antigen or TGF-β. On days 5 through 9, concentrations of 1 and 10 µg/ml PC61 nearly saturated CD25 as determined by a lack of surface labeling by an APC-conjugated PC61 mAb (*y*-axis), because pre-existing PC61–CD25 complexes prevented the binding of APC-conjugated PC61. These PC61 concentrations (1 and 10 µg/ml) respectively facilitated Treg enrichment to frequencies of 45 and 74%. PC61 dose dependently augmented GFP MFI (mean fluorescence intensity; four-digit number on the right of right-most column) on day 9 and thereby supported the hypothesis that PC61 preferentially selected mature Tregs. In the presence of 0.1 µg/ml PC61 or in control cultures without PC61, Tregs and Tcons expressed high levels of free CD25, and Treg frequencies were sparse at approximately 20% throughout the 9 days of culture. Importantly, PC61 by itself did not induce FOXP3, as T cells activated without TGF-β and cultured in the presence of PC61 did not result in Tregs (Figure 2D). These data indicate that TGF-β-iTregs continuously required saturating PC61 concentrations during propagation in IL-2 to engender high percentages of Tregs.

**Figure 3:**
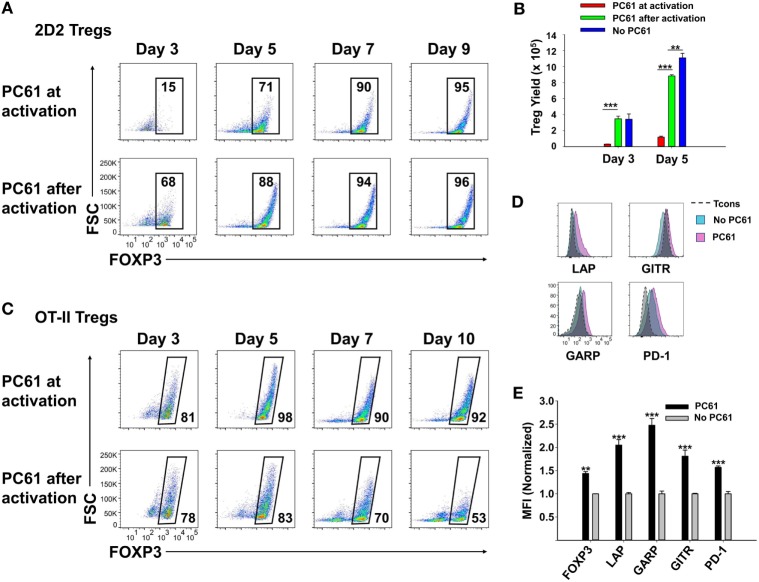
PC61 allowed for rapid generation of pure regulatory T cell (Treg) lines. 2D2-FIG splenocytes (SPL) **(A,B,D,E)** or OTII-FIG SPL **(C)** were activated at a density of 2 × 10^6^ cells/ml in cRPMI with MOG35-55 (1 µM) or 100 nM OVA323-339, respectively, with 10 nM TGF-β in the presence or absence of 10 µg/ml PC61. Antigen and TGF-β were present only in the initial 3-day activation culture and not thereafter. **(A–C)** PC61 was (PC61 at activation) or was not (PC61 after activation) included in the initial 3-day activation culture but was included in both groups after passage of the cells on day 3. Cells were counted and then analyzed to determine percentages of viable, Vβ11^+^ (2D2), Vβ5.1^+^ (OTII), FOXP3^+^ T cells on the designated days. **(B)** Shown are the 2D2-FIG Treg yields on days 3 and 5. **(D,E)** After 3 days of activation with or without PC61, 2D2-FIG T cells were passaged for 10 days at a density of 10^6^ cells/ml in rat IL-2 in the presence or absence of 10 µg/ml PC61. Cells gated as Vβ11^+^ FOXP3^+^ Tregs or FOXP3^−^ conventional T cells (Tcons) were analyzed for expression of latency-associated peptide (LAP), GARP, GITR, and PD-1 on day 10 post-activation. MFI Values were normalized so that values obtained from T cells cultured in the absence of PC61 was equal to 1. These experiments are representative of three independent experiments.

Although PC61 stabilized Tregs in IL-2 maintenance cultures, inclusion of PC61 in the initial 3-day activation culture appeared to delay Treg induction. To assess this issue, PC61 was or was not included in the initial 3-day activation culture together with 2D2-FIG SPL, MOG35-55, and TGF-β (Figure 3A), and then PC61 was included in the IL-2 maintenance cultures of both groups through day 9. Regardless of whether PC61 was added on day 0 (PC61 at activation) or day 3 (PC61 after activation), PC61 facilitated the emergence of highly enriched FOXP3^+^ Tregs by day 9. However, introduction of PC61 after the initial activation culture was advantageous because this regimen resulted in the accelerated enrichment of Tregs as noted by differences at days 3–5 (Figure 3A) and increased Treg yields at day 5 (Figure 3B). Treg yield on day 5 post-activation was more than sevenfold higher in the “PC61 after activation” group compared to “PC61 at activation” group. Cultures that lacked PC61 in both phases of culture (No PC61) resulted in a dramatic reduction in Treg percentages but similar cell yields compared to the “PC61 after activation” group. Interestingly, PC61 was necessary at activation and during propagation to generate highly pure, stable OT-II Treg lines (Figure 3C). This phenomenon is most likely due to the high responsiveness of OT-II T cells to their cognate antigen (OVA323-339). That is, PC61-mediated inhibition was needed earlier in the activation cascade due to the higher antigen reactivity of OT-II T cells. Thus, the requirement for the anti-CD25 mAb during the initial culture for OTII-FIG T cells may reflect the antigenic potency of the cognate antigen. Nevertheless, 2D2 and OT-II FOXP3^+^ enriched lines were obtained without physical purification or any genetic modification by the end of the experiment. These findings indicated that the presence of PC61 during the IL-2 expansion phase was critical for achieving high Treg percentages in stable FOXP3^+^ Treg lines.

To assess whether PC61 affected expression of selected Treg markers, 2D2-FIG SPL were subjected to a 3-day activation with MOG35-55 and TGF-β (Figures 3D,E). The T cells were then passaged every 3–4 days in IL-2 for an additional 10 days in the presence or absence of PC61, whereby the lines were almost exclusively small, rested T cells. Gated Treg and Tcon subsets were analyzed separately. PC61-cultivated Tregs differed from control (no PC61) cultures *via* higher Treg expression of FOXP3, LAP, GARP, GITR, and PD-1. These data revealed that PC61-maintained Tregs had markers consistent with the Treg lineage and expressed significantly higher levels of these markers compared to Tregs cultivated in the absence of PC61, signifying the ability of PC61 to select mature Tregs.

**Figure 4:**
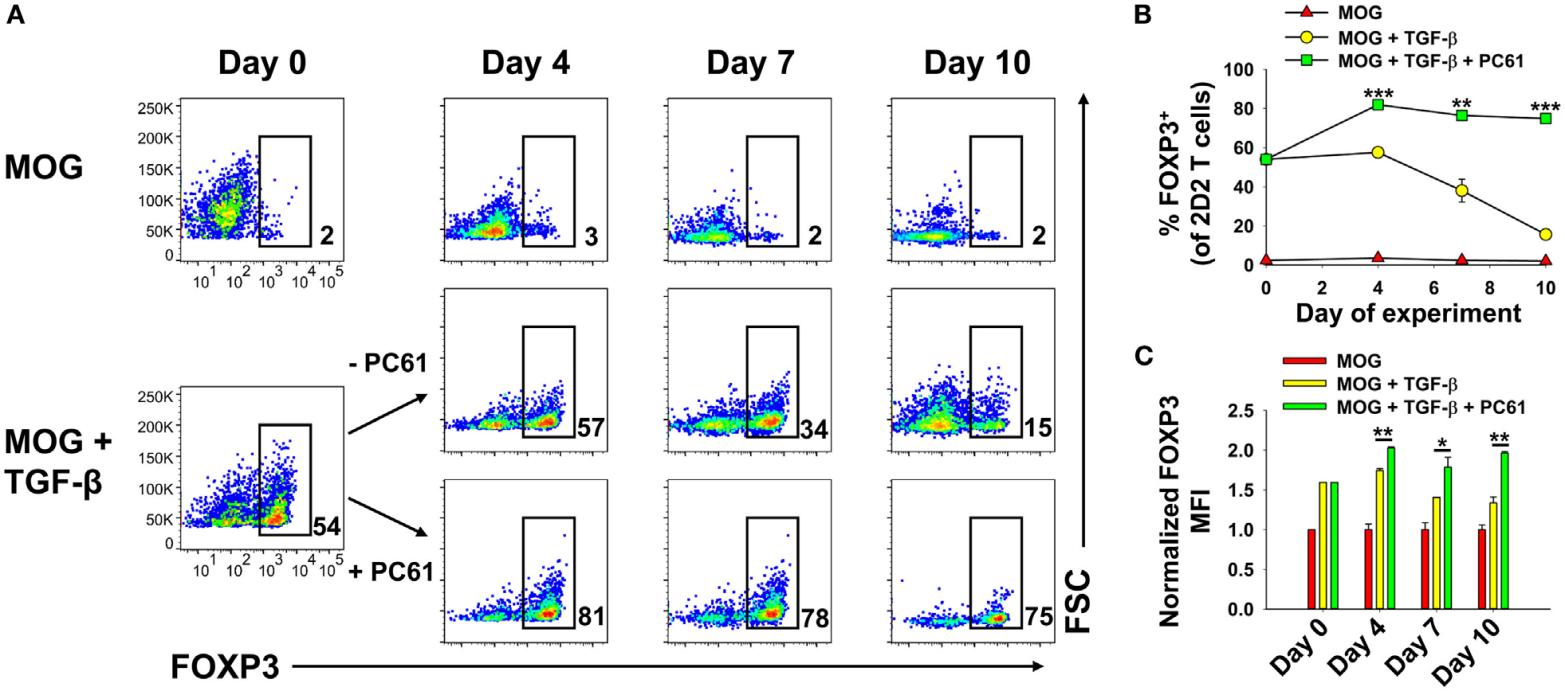
PC61 enabled the preferential outgrowth of TGF-β-induced iTregs from 2D2-FIG Rag1^−/−^ mice. 2D2-FIG Rag1^−/−^ mice splenocytes were activated for 4 days at a density of 2 × 10^6^ cells/ml in cRPMI with MOG35-55 in the presence or absence of 1 nM TGF-β to generate regulatory T cells (Tregs) and conventional T cells, respectively, and were passaged at a density of 5 × 10^5^ cells/ml in cRPMI with rat IL-2 on day 0 of the rest phase. T cells were then passaged with or without PC61 (10 µg/ml) (in the absence of TGF-β and MOG) as designated every 2–4 days. Vβ11^+^ T cells were assessed for FOXP3 expression on days 0, 4, 7, and 10 after activation. Shown are representative dot plots of the percentage of FOXP3^+^ Tregs in the Vβ11^+^ T cell population **(A)** and the corresponding averages of triplicate samples **(B)**. The bar graph shows the geometric MFI of FOXP3 expression in the FOXP3^+^ gate **(C)**. The geometric mean fluorescence intensity was normalized so that the FOXP3 MFI of Tregs activated in the absence of TGF-β was equal to 1. *p < 0.05, **p < 0.01, ***p < 0.001. These experiments are representative of three independent experiments.

An important question was whether iTregs that were induced *de novo* during activation in the presence of TGF-β were stabilized by PC61, because iTregs may be important starting material for derivation of antigen-specific Tregs. Previous experiments however were initiated with total 2D2-FIG SPL, which may include pre-existing thymically derived tTregs that may contribute or dominate growth in the presence of PC61. In 2D2-FIG mice, pre-existing Tregs typically comprise less than 1% of CD4^+^ T cells, with a frequency range of approximately 0.2–1.5% pre-existing FOXP3^+^ Tregs. To assess this issue, Tregs were induced with MOG and TGF-β from 2D2-FIG *Rag1^−^*^/^*^−^* mice, which lack tTregs (Figure 4). 2D2-FIG *Rag1^−^*^/^*^−^* Tregs were cultured in the presence of IL-2 with or without PC61. Tregs cultured in the presence of PC61 showed a stable, high percentage of Tregs (>75%), whereas Tregs cultured in the absence of PC61 showed a diminishing Treg percentage throughout the duration of the experiment (Figures 4A,B). The FOXP3 geometric mean fluorescence intensity (gMFI) of Tregs cultured in the presence of PC61 was significantly higher than the gMFI of Tregs cultured in the absence of PC61 (Figure 4C). These data provide evidence that PC61 stabilizes outgrowth of TGF-β-induced iTregs.

### PC61 Was the Anti-CD25 mAb of Choice for Blockade of IL-2 Signaling and Selection of Tregs

**Figure 5:**
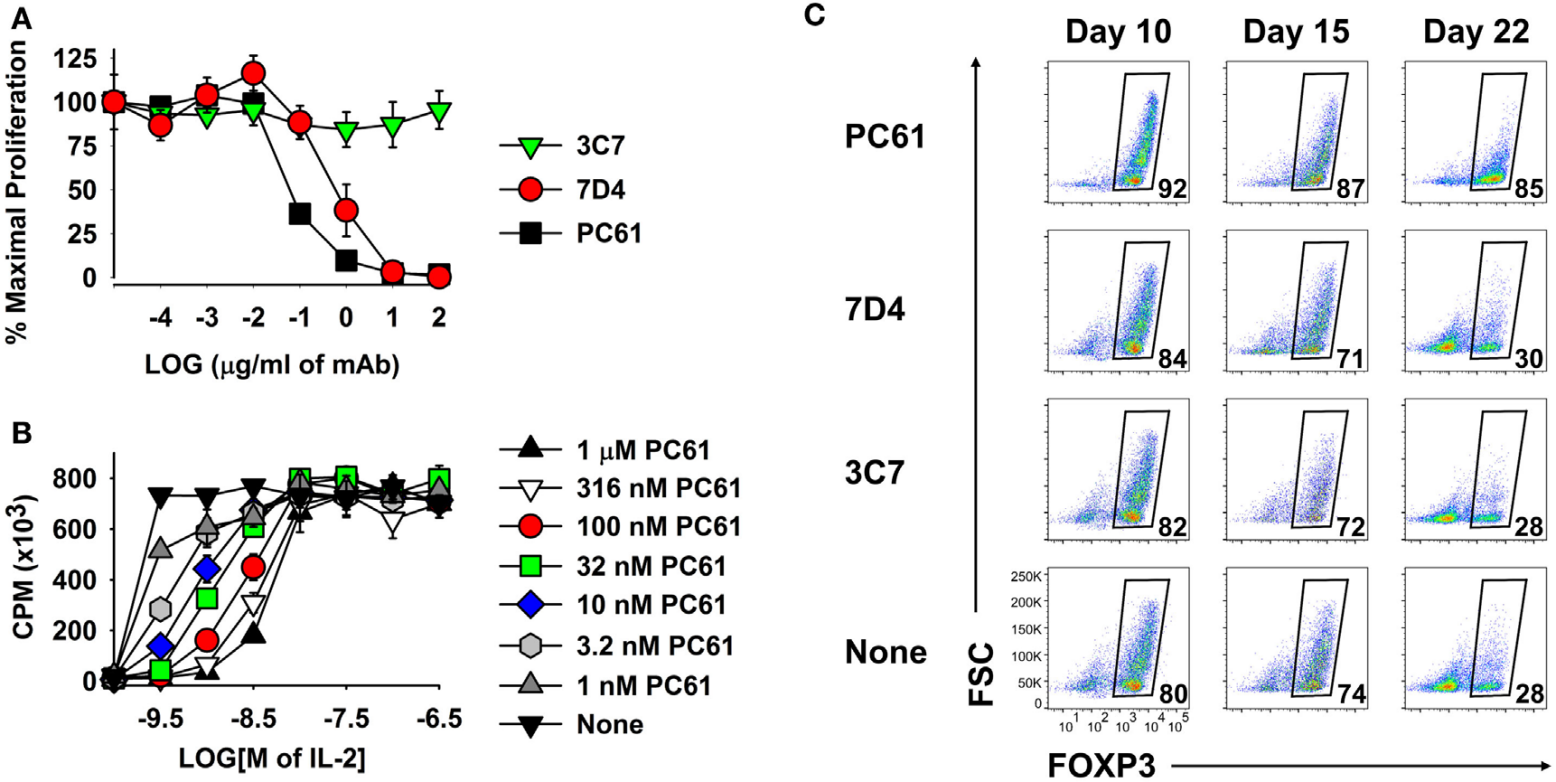
The PC61 anti-CD25 monoclonal antibody (mAb) was a functional antagonist of IL-2 growth at low concentrations of IL-2 and was superior to the 7D4 and 3C7 anti-CD25 mAbs for maintenance of regulatory T cells. **(A,B)** A continuously propagated line of IL-2 dependent CD4^+^ T cells (SJL-PLP.1 T cells) was used as IL-2 responders in these assays. These T cells (3,000/well) were cultured with rat IL-2 and designated concentrations of PC61, 7D4, or 3C7 mAbs **(A)** or were cultured with designated concentrations of purified PC61 and mouse IL-2 **(B)**. Cells were pulsed with [^3^H]thymidine during the last 24 h of a 3-day culture. **(C)** 2D2-FIG splenocytes were activated at a density of 2 × 10^6^ cells/ml cRPMI with 1 µM MOG35-55 in the presence of 10 nM TGF-β. After 3 days, cells were passaged at a density of 10^6^ cells/ml with IL-2 plus 10 µg/ml PC61, 7D4, 3C7, or no anti-CD25 mAb. Cells were passaged every 3–4 days in IL-2 with the same anti-CD25 antibody. Vβ11^+^ T cells were analyzed for FOXP3 expression on days 10, 15, and 22. These experiments are representative of three independent experiments.

Because anti-CD25 mAbs differ in epitope specificity and inhibitory mechanism, three anti-CD25 mAb were screened for inhibitory efficacy in assays of IL-2-dependent T cell growth. The PC61 mAb (rat IgG1, λ) was more suppressive than the 7D4 mAb (rat IgM, κ), whereas the 3C7 mAb (rat IgG2b, κ) lacked inhibitory activity at this concentration of IL-2 (Figure 5A). PC61 may have superior efficacy because PC61 and IL-2 bind distinct sites on the CD25 IL-2Rα, and PC61 facilitates the dissociation of IL-2, but not *vice versa* (31). Although PC61 is routinely used for depletion of Tregs *in vivo* (27), the PC61 mAb had no cytolytic or depleting activity *in vitro*, as shown previously in Figure 2. PC61 mediates Treg depletion *in vivo via* FcγR-mediated clearance (27), but this mechanism was not operational in T cell cultures.

As shown in Figure 5B, PC61 and IL-2 exhibited functional competition in cultures of a transformed Tcon line that expressed a CD25^high^ phenotype and potent IL-2 responsiveness. High concentrations of PC61 (up to 1 µM) inhibited IL-2 responses in the presence of limiting IL-2 concentrations (<10 nM) as shown by right-shifted IL-2 concentration–response curves. In contrast, high concentrations of IL-2 (≥10 nM) overwhelmed the inhibitory action of all tested concentrations of PC61. High concentrations of IL-2 may signal through lower affinity IL2Rβ–γ complexes and thereby may drive IL-2 dependent growth independent of CD25. Overall, these data indicate that PC61 effectively inhibited IL-2 responses, but only within a range of relatively low IL-2 concentrations.

As predicted based on its superior IL-2 inhibitory activity (Figure 5A), PC61 was more efficient than the anti-CD25 mAb 7D4 and 3C7 for maintenance of Treg cultures (Figure 5C). Due to the superior inhibitory efficacy and superior Treg selectivity, the anti-CD25 PC61 mAb was used for the remainder of the study.

### PC61 Enabled the Dominant Outgrowth of Tregs in Mixed Treg/Tcon Cultures

**Figure 6:**
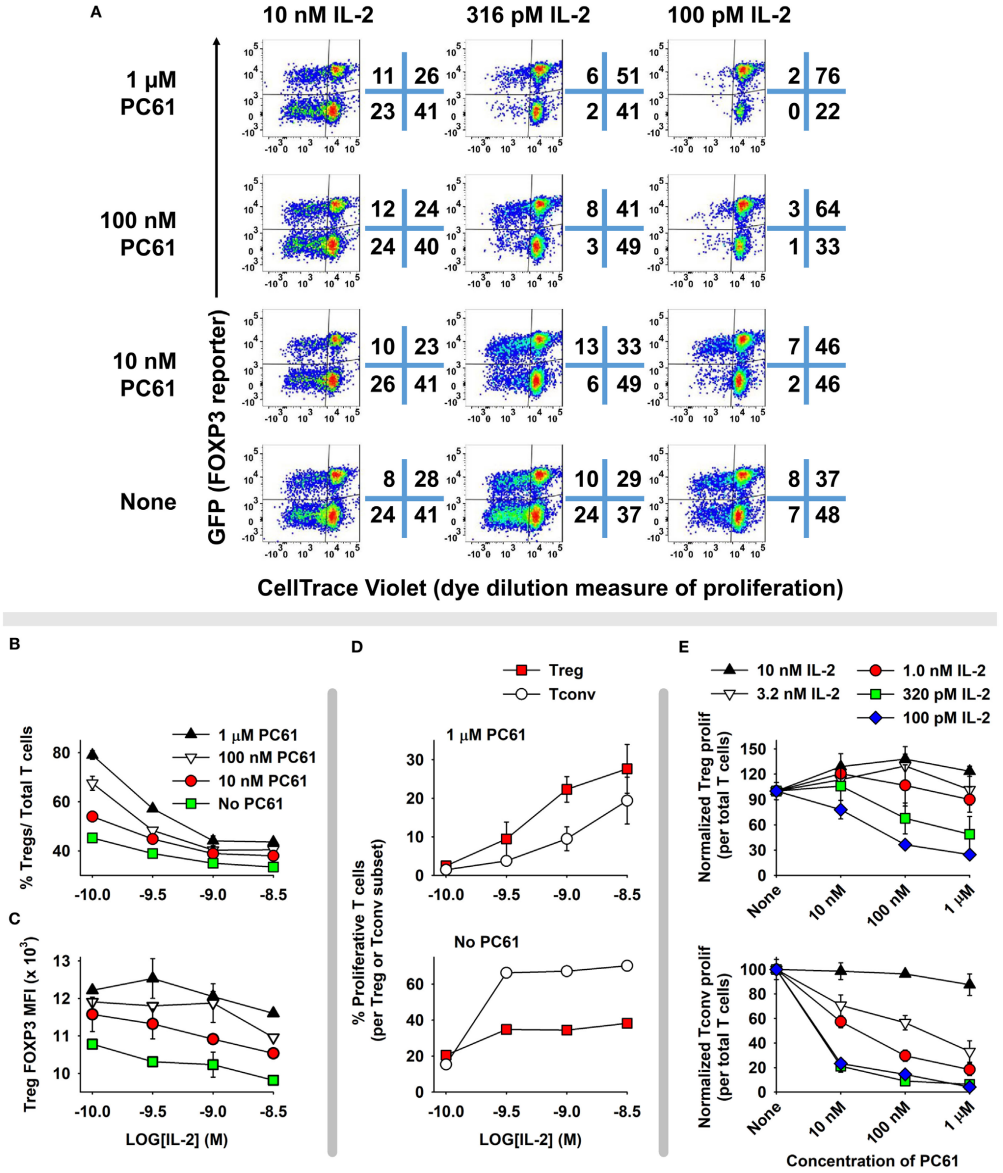
A permissive “Treg window” that favored dominance of FOXP3^+^ regulatory T cells (Tregs) was defined by low IL-2 concentrations and high PC61 concentrations. 2D2-FIG T cells were cultured with 1 µM MOG35-55 for 4 days in the presence or absence of 10 nM TGF-β to establish lines of FOXP3^+^ Tregs and FOXP3^−^ conventional T cells (Tcons), respectively. T cells were passaged every 3 or 4 days for a total 16 days in IL-2, and Treg cultures were also supplemented with 10 µg/ml PC61 to select and establish high percentages of FOXP3^+^ Tregs. At the initiation of the experiment, Tregs and Tcons were equally mixed and labeled with CTV and were cultured for 6 days in designated concentrations of IL-2 (100 pM–10 nM) in the presence or absence of designated concentrations of PC61. Dotplots in panel **(A)** show proliferating (left quadrants) versus non-proliferating (right quadrants) Tregs (top quadrants) and Tcons (bottom quadrants). Shown are percentages **(B)** and MFI **(C)** of FOXP3^+^ Tregs in the Vβ11^+^ population. **(D)** Shown are the percentages of proliferating FOXP3^+^ Tregs and FOXP3^−^ Tcons in the presence or absence of 1 µM PC61. **(E)** Normalized proliferation of Tregs and Tcons at designated concentrations of IL-2 and PC61. This experiment is representative of three independent experiments.

The concept of a Treg window was tested with mixed 2D2 (MOG-specific) lines of FOXP3^+^ and FOXP3^−^ T cells (Figure 6). These T cells were propagated in IL-2 for 16 days before use in this assay, so that Tregs represented nearly 96% of the T cells in the FOXP3^+^ line. Equal numbers of Tcons and Tregs were labeled with CellTrace Violet (CTV) and were cultured for 6 days with designated concentrations of IL-2 and PC61. This analysis distinguished proliferative subsets (left quadrants) from non-proliferative subsets (right quadrants) as well as Tregs (upper quadrants) from Tcons (lower quadrants) (Figure 6A). Shown are two relatively low IL-2 concentrations (100 and 316 pM) in which proliferative and non-proliferative Tregs were dominant (exhibited higher percentages) compared to Tcons when cultured with 1 µM PC61. At 100 pM IL-2 and either 1 µM PC61 or “no PC61,” 78 or 45% of T cells (sum of upper two quadrants) were Tregs, respectively. At 316 pM IL-2, proliferative Tregs exhibited higher frequencies than proliferative Tcons in the presence but not absence of 10 nM, 100 nM, and 1 µM PC61. At high IL-2 concentrations (e.g., 10 nM), Tcons were dominant and showed overgrowth regardless of PC61 concentration. The concentration-dependent ability of PC61 to promote Treg dominance in low IL-2 concentrations (Figure 6B) correlated with preservation of high GFP fluorescence (Figure 6C), which is an indirect measure of FOXP3 expression on a per cell basis. Tregs were dominant within a 10-fold range of IL-2 concentrations (320 pM–3.2 nM, Figure 6D) in the presence of 1 µM PC61, whereas Tcons were dominant at the same IL-2 concentrations in the absence of PC61. Although PC61 exhibited concentration-dependent inhibition of IL-2-dependent Treg growth, PC61 was a more potent inhibitor of Tcon growth at limiting IL-2 concentrations, such that differential growth and survival favored persistence of Tregs over Tcons (Figure 6E). Overall, these data reveal a “Treg window” defined by ranges of IL-2 and PC61 concentrations that enabled dominant survival and outgrowth of FOXP3^+^ Tregs.

### The Anti-CD25 PC61 Antibody Enabled Maintenance of Long-term Lines of FOXP3^+^ Tregs

**Figure 7:**
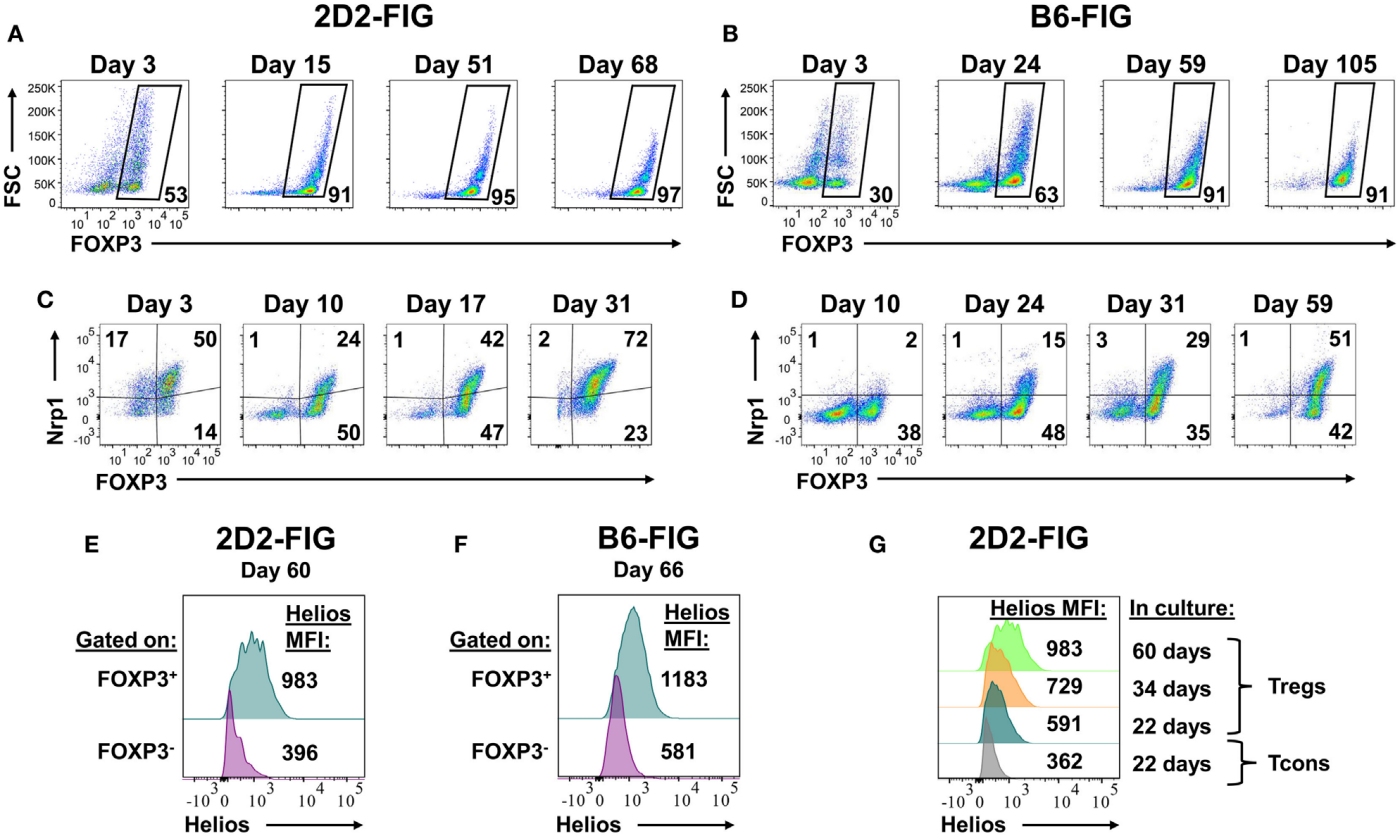
The anti-CD25 monoclonal antibody PC61 enabled long-term, stable propagation of FOXP3^+^ regulatory T cells (Tregs). 2D2-FIG splenocytes (SPL) or FIG SPL were activated with 1 µM MOG35-55 or 2.5 µg/ml Con-A, respectively, at a density of 2 × 10^6^ SPL/ml in the presence of 10 nM TGF-β. After 3 days of activation, cells were passaged at a density of 10^6^ cells/ml in media containing rat IL-2 and 10 µg/ml PC61. Cells were passaged every 3–4 days in the same conditions for the indicated durations. In 2D2-FIG cultures, Vβ11^+^ cells were analyzed on the indicated days for FOXP3 expression **(A)**, Neuropilin-1 expression **(C)**, and Helios expression **(E,G)**. Panels **(A,C)** represent analyses of lines established from separate mice. **(B,D,F)** In C57BL/6 (B6)-FIG cultures, CD4^+^ T cells were purified to remove CD8^+^ T cells. FIG lines were analyzed for FOXP3 expression **(B)**, Neuropilin expression **(D)**, and Helios expression **(F)**. For panels **(E,F)**, Helios expression was measured in the FOXP3^+^ and the FOXP3^−^ populations from the same line. These experiments are representative of three independent experiments.

Given that PC61 was instrumental in selecting enriched FOXP3^+^ Treg lines over a 2- to 3-week timespan (Figure 2), a critical question was whether PC61 also allowed maintenance of highly enriched Treg lines over the course of months. To assess this question, 2D2-FIG SPL were activated with MOG35-55 and TGF-β in a 3-day activation in the absence of PC61 and were then passaged every 3–4 days in IL-2 with 10 µg/ml PC61 (Figure 7A). Over the course of 68 days, the line was analyzed for percentages of FOXP3^+^ Tregs. Tregs represented over 90% of the cells by day 15 and persisted at these levels throughout the duration of the culture without showing any signs of attenuation. Equivalent lines propagated in IL-2 but in the absence of PC61 showed progressive loss and extinction of FOXP3^+^ Tregs across 2–4 weeks of propagation in IL-2 (e.g., Figure 2).

FOXP3^+^ Treg lines from naïve, clonotypically diverse FIG SPL could also be derived by addition of PC61 to IL-2 expansion cultures (Figure 7B). Naïve FIG SPL were activated for 3 days with Con-A and TGF-β and then were propagated in IL-2 in the presence of PC61. CD4^+^ T cells were purified to remove CD8^+^ T cells in polyclonal FIG SPL cultures to prevent CD8^+^ T cell overgrowth of the line, but CD4^+^ T cell purification was not necessary for 2D2-FIG Tregs given that 2D2-FIG mice largely lacked CD8^+^ T cells. Over the course of 105 days, cultures became progressively enriched with polyclonal FOXP3^+^ Treg cells until over 90% of the line was comprised of FOXP3^+^ Tregs. In subsequent polyclonal FIG Treg lines, CD4 purification was performed on day 4 post-activation as opposed to day 10. This earlier intervention allowed for the generation of Treg lines consisting of >90% Tregs by day 10 (not shown). Culture of Treg lines in IL-2 expansion cultures with PC61 resulted in a slow and steady expansion of cell numbers. These findings reveal the generalized applicability of PC61-mediated Treg selection in mice. The implication is that Treg lines can be generated and maintained indefinitely in the presence of PC61. We have generated at least 15 different 2D2-FIG Treg lines, 5 different OT-II Treg lines, and 4 different polyclonal Treg lines and carried them for more than 50 days with Treg percentages greater than 85% of the T cell population. Overall, these lines showed no loss of Treg percentages and no decrement in GFP fluorescence (i.e., FOXP3 expression) over the entire culture duration.

These long-term lines expressed Neuropilin-1 and Helios, which have been associated with the FOXP3^+^ Treg phenotype (Figures 7C–G). Neuropilin-1 was expressed on both 2D2-FIG Tregs (Figure 7C) and polyclonal Tregs (Figure 7D). Neuropilin-1 exhibited progressive increases in MFI as a function of time such that Neuropilin-1 expression was positively correlated with the longevity of culture. Given that Helios is a transcription factor implicated in Treg function, we also analyzed 2D2-FIG Tregs on day 60 of culture (Figure 7E) and polyclonal Tregs on day 66 of culture (Figure 7F) for expression of Helios. 2D2-FIG Tregs and polyclonal Tregs both expressed Helios, whereas Tcons lacked detectable expression. Like Neuropilin-1, Helios expression levels correlated with the duration of culture (Figure 7G). These findings provided evidence that expression of both Neuropilin-1 and Helios was amplified by continuous, low-intensity IL-2 signaling in continuity with Treg lineage commitment.

### Reactivation and Expansion of FOXP3^+^ Treg Lines

**Figure 8:**
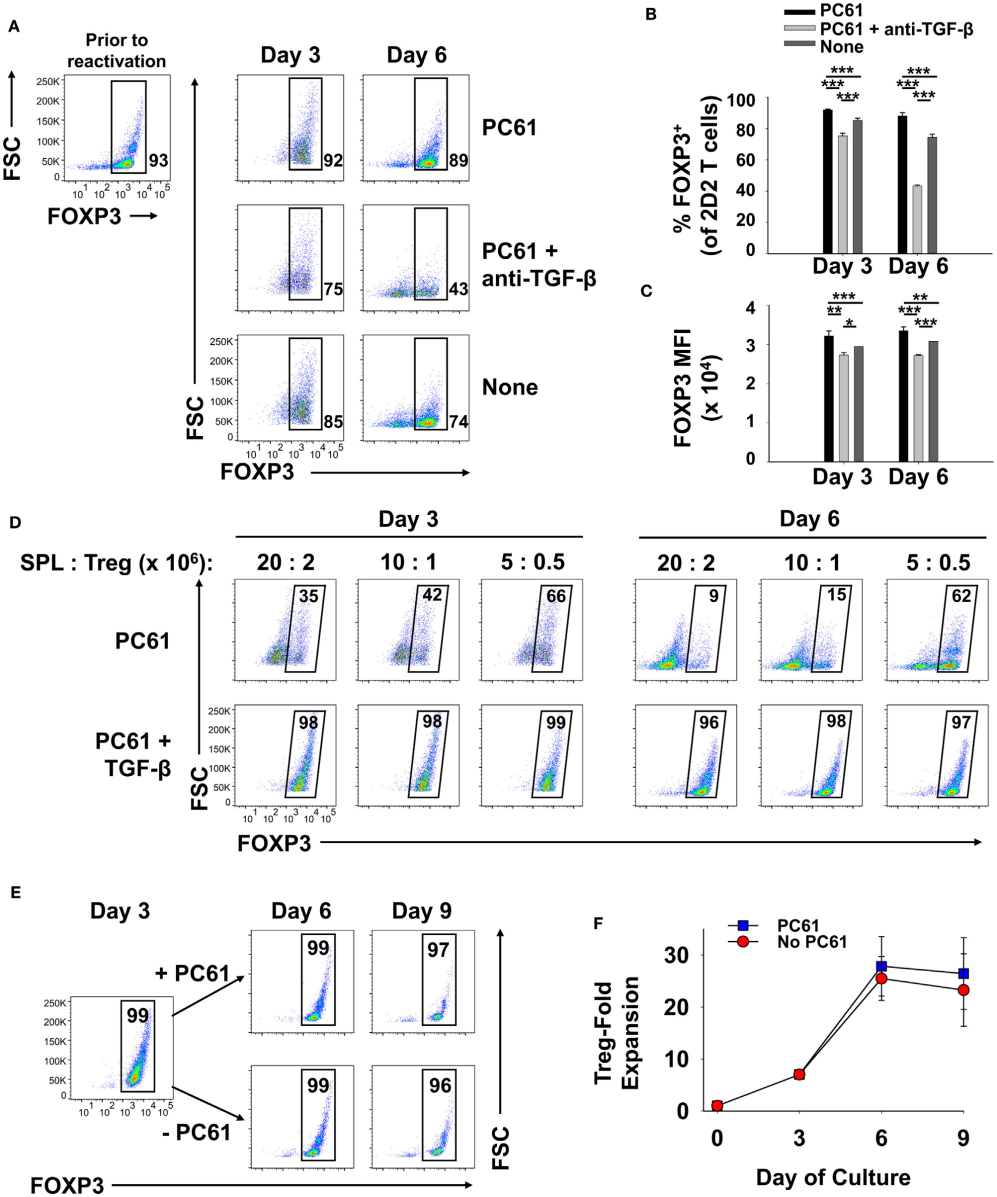
TGF-β was required for the maintenance of regulatory T cell (Treg) stability during secondary Treg activation and expansion. **(A–C)** 100,000 Tregs (rested for 23 days, 93% FOXP3^+^) and 10^6^ irradiated splenic APC were cultured in 200 µl with 1 µM MOG35-55 and IL-2 with or without 10 µg/ml PC61 and the 1D11 anti-TGF-β monoclonal antibody (50 µg/ml) as designated. Cells were analyzed on days 3 and 6 of activation. **(A)** FOXP3^+^ Treg percentages are shown in bottom right corner for the Vβ11^+^ 2D2 TCR-gated population. Also shown are the average percentages of FOXP3^+^ Tregs per Vβ11^+^ T cells **(B)** and average FOXP3 (GFP) MFI **(C)**. Error bars represent SDs. *p < 0.05, **p ≤ 0.01, ***p ≤ 0.001. **(D)** Tregs that were rested for 33 days (94% FOXP3^+^) were reactivated with irradiated splenocytes (SPL) and 1 µM MOG35-55 in the presence of 10 µg/ml PC61 with (bottom row) or without (top row) 100 pM TGF-β. Activation cultures contained rat IL-2 with designated densities of Tregs and irradiated splenic APC in 5 ml media as indicated above each set of dotplots. For example, 20 × 10^6^ irradiated SPL were used to activate 2 × 10^6^ Tregs. Alternatively, 10 × 10^6^ or 5 × 10^6^ APC were used to activate 10^6^ or 5 × 10^5^ Tregs, respectively, in a 5-ml volume. Cells were analyzed on days 3 and 6 of activation by gating on Vβ11^+^ T cells. Percentages of FOXP3^+^ cells are shown in the top right corner of each dotplot. **(E,F)** 250,000 2D2 induced-Tregs were induced by culturing FACS-sorted naïve FOXP3^null^ T cells with 50,000 irradiated bone marrow-derived dendritic cells, 2.5 µg/ml Con-A, IL-2, and 1 nM TGF-β. T cells were passaged into IL-2 with or without 10 µg/ml PC61 on day 3 and were subsequently passaged again on day 6 at a density of 5 × 10^5^ cells/ml. Cells were counted by Trypan Blue exclusion and analyzed by flow cytometry for FOXP3 expression on Vβ11^+^ gated T cells. Cell yields **(F)** were calculated by multiplying cell counts by the percentage of Vβ11^+^ FOXP3^+^ cells on days 3, 6, and 9. These experiments are representative of three independent experiments.

We assumed that reactivation of FOXP3^+^ Treg lines would be a necessity for Treg-mediated suppressive activity in adoptive transfer experiments. The critical issue was whether FOXP3^+^ Tregs could undergo secondary activation *in vitro* without loss of FOXP3 and the Treg phenotype. Like the primary activation, we hypothesized that reactivation of established Treg lines would be contingent on TGF-β. To assess this issue, a 2D2-FIG line (>90% FOXP3^+^ Tregs) that had been cultured for 23 days in IL-2 and PC61 was reactivated for 3 or 6 days with irradiated splenic APC and MOG35-55 in the presence of IL-2 with or without PC61 and the neutralizing anti-TGF-β 1D11 mAb (Figures 8A–C). The presence of PC61 during secondary activation stabilized Treg percentages at approximately 90% (Figure 8A top row; Figure 8B). The presence of PC61 also stabilized FOXP3 expression, as reflected by GFP MFI (Figure 8C). Notably, 1D11-mediated neutralization of TGF-β resulted in a loss of FOXP3^+^ Treg percentages to less than 50% and a decrement in FOXP3 expression on a per cell basis, as reflected by the GFP MFI (Figure 8A middle row; Figure 8C). These data indicate that FOXP3 expression during antigen-driven cellular activation was maintained by TGF-β produced by cells in the culture.

These data provided evidence that exogenous TGF-β might augment Treg stability in subsequent reactivation cultures. To assess this issue, designated cell densities were used at a constant 10:1 APC:Treg ratio at 4 × 10^5^ Tregs/ml, 2 × 10^5^ Tregs/ml, or 10^5^ Tregs/ml (Figure 8D; Table 1). In the absence of exogenous TGF-β, T cells underwent activation and expansion at each density (Table 1), but Treg percentages declined, particularly at higher cell densities. Most likely, high T cell densities caused a more robust activation and perhaps enhanced the production of pro-inflammatory cytokines (i.e., IL-23 or GM-CSF) that might antagonize the action of TGF-β. Notably, the presence of exogenous TGF-β prevented decrement of Treg frequencies and stabilized the Treg phenotype regardless of the cell density. Based on cell counts, high initial cell densities in the presence of TGF-β enabled the most robust expansion of Tregs by day 3 of activation (Table 1). These data indicate that optimal Treg expansion requires the presence of exogenous TGF-β during secondary activation. This observation is key to strategies for expansion of continuous FOXP3^+^ Treg lines.

**Table 1:** Regulatory T cells (Tregs) exhibit stable expansion in the presence of exogenous TGF-β, IL-2, and PC61.

Group^a^	APC: Treg # (×10^6^) on day 0	Cell # (×10^6^) on day 3	Treg # (×10^6^) on day 3	Treg yield^b^	FOXP3^+^ Treg percentages
No TGF-β	20:2	9.58	3.40	1.7	35
	10:1	3.33	1.44	1.4	42
	5:0.5	2.20	1.48	3.0	66
TGF-β	20:2	13.36	13.13	6.6	98
	10:1	5.26	5.18	5.2	98
	5:0.5	2.16	2.13	4.3	99

*^a^T cells were rested for 33 days and were 94% FOXP3^+^ Tregs at the initiation of the experiment. T cells were activated with irradiated splenocytes (SPL), 1 µM MOG35-55, rat IL-2, and 10 µg/ml PC61 and in the presence or absence of 100 pM TGF-β. Cultures were setup in 5 ml media containing designated numbers of Tregs and irradiated SPL (column 2)*.

*^b^Cells were analyzed on day 3 of activation by gating on Vβ11 and FOXP3 (GFP). Absolute cell counts (Trypan Blue exclusion), Vβ11^+^ T cell percentages, and FOXP3^+^ Treg percentages were used to calculate Treg numbers. Treg yield was calculated by dividing the Treg cell count on day 3 by the starting Treg count (2 × 10^6^, 1 × 10^6^, or 0.5 × 10^6^) on day 0. These data are representative of three independent experiments*.

To quantitate Treg expansion, an established, long-term 2D2-FIG Treg line was activated for 3 days with irradiated bone marrow-derived dendritic cells, 2.5 µg/ml Con-A, 1 nM TGF-β, and IL-2 (Figures 8E,F). T cells were then passaged on days 3 and 6 at a density of 5 × 10^5^ cells/ml cRPMI containing rat IL-2 with or without PC61. Viable cells were enumerated (Trypan Blue dye exclusion) and analyzed for Vβ11 and FOXP3 expression to calculate expansion of viable FOXP3^+^ Tregs. During the antigenic activation (days 0–3), Tregs expanded ~7-fold while retaining high FOXP3 expression in nearly 100% of the population. Tregs expanded another ~4-fold from days 3 to 6 for a net 26- to 28-fold expansion since day 0 (Figure 8F). Importantly, there was no difference in the expansion rates between Tregs cultured in the presence or absence of PC61 immediately following antigenic activation, such that CD25^high^ Tregs were resistant to PC61 antagonism. From days 6 to 9, Tregs reverted to a quiescent resting phase and did not expand in numbers either with or without PC61. The percentage of FOXP3^+^ Tregs in each culture remained >98% through the 6 days of IL-2 propagation regardless of the presence or absence of PC61. Overall, in long-term cultures of resting quiescent Tregs (i.e., weeks to months without activation), cell numbers were static or modestly expanded (onefold to twofold) over the course of a week. These data provide evidence that 2D2 Tregs can undergo an approximate 26-fold expansion over the course of 9 days following a 3-day cellular activation and 6 days of rest. This activation–rest strategy is based on the classical method for derivation of T cell lines and is also sufficient for expansion of stable FOXP3^+^ Treg lines.

Although PC61 was deleterious in the initial activation culture (Figure 2A), PC61 was beneficial in the secondary reactivation cultures (Figure 8). However, this difference reflected a difference of strategy regarding the initial selection of the line versus the subsequent reactivation and expansion of the line. That is, exogenous IL-2 was not added to primary activations to reinforce antigen specificity of the initial activation, whereas optimization experiments showed that the inclusion of exogenous IL-2 in secondary reactivation promoted expansion of the established clonotypic line. Basically, PC61 preserved the FOXP3 phenotype whenever exogenous IL-2 was added to the culture because unmitigated IL-2 signaling resulted in Tcon dominance and overgrowth/destabilization of the Treg line. Thus, PC61 was beneficial in secondary reactivation cultures because these cultures were supplemented with exogenous IL-2.

### Cultured Tregs Exhibited *In Vitro* and *In Vivo* Suppressive Activities

**Figure 9:**
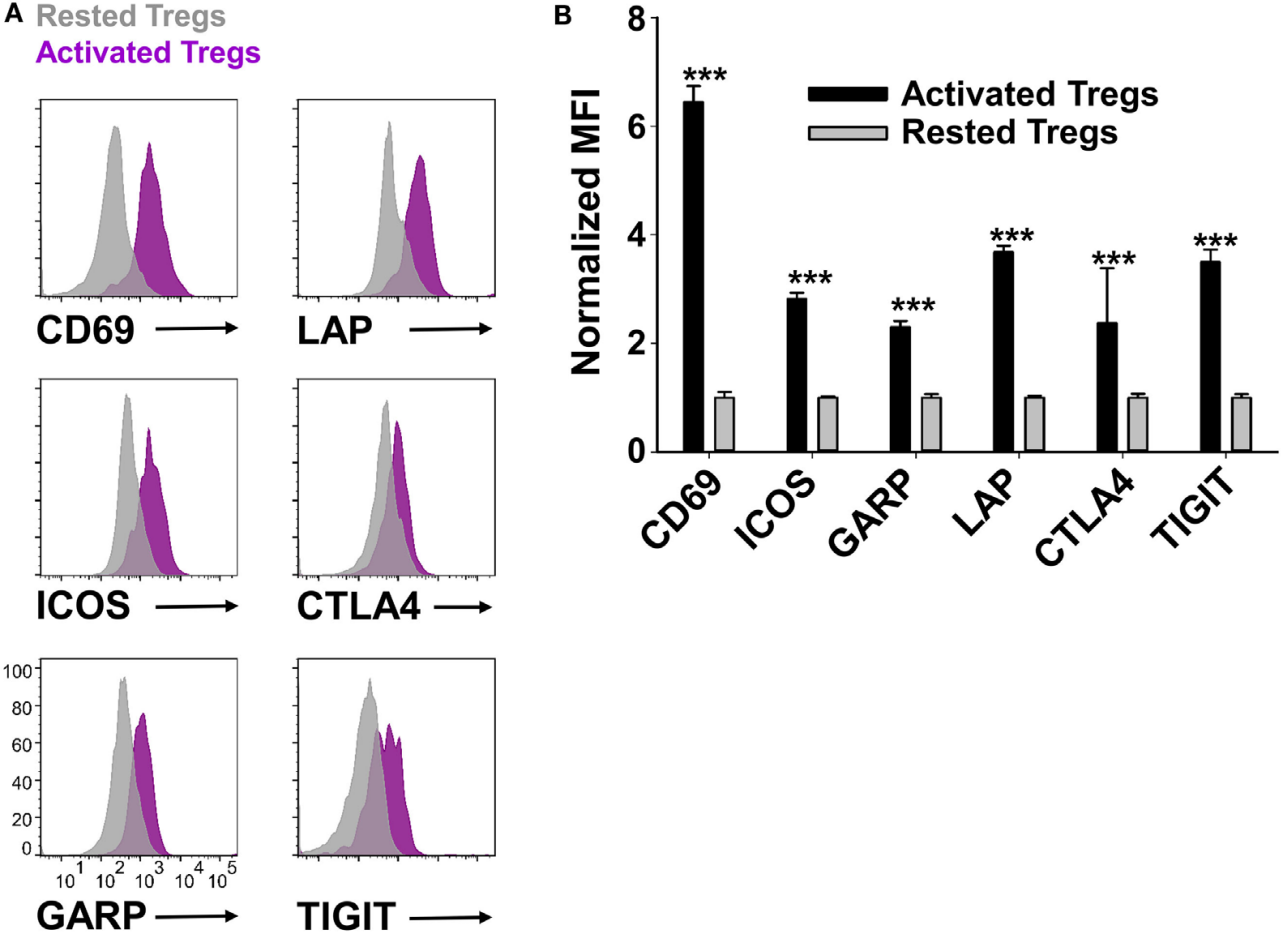
Reactivated regulatory T cells (Tregs) upregulated Treg-associated activation markers. To generate activated Tregs, 100,000 rested Tregs (>90% FOXP3^+^) and 10^6^ irradiated splenocytes were cultured for 2 days with 1 µM MOG35-55, rat IL-2, and 10 µg/ml PC61. A control rested Treg line was passaged in IL-2 and PC61. Activated Tregs versus rested FOXP3^+^ Tregs were gated and analyzed for the indicated markers. Representative overlapping histograms are shown in panel **(A)**. Relative MFIs for the indicated markers on gated Tregs are shown in panel **(B)**. MFI values were normalized so that rested Treg MFI values were equal to 1. ***p < 0.001. These data are representative of three independent experiments.

**Figure 10:**
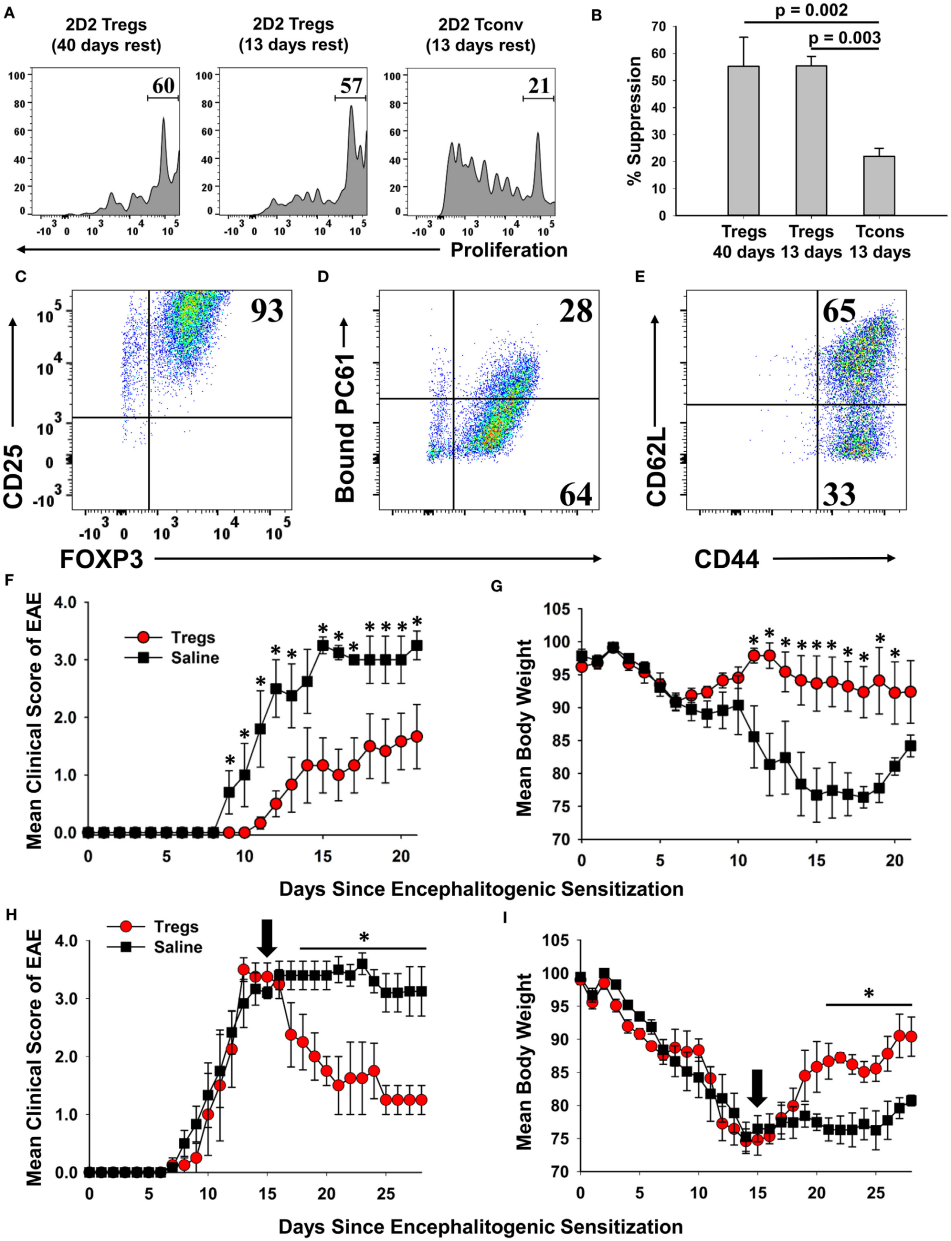
A 2D2-FIG, MOG-specific FOXP3^+^ regulatory T cell (Treg) line exhibited in vitro and in vivo suppressive activities. **(A,B)** To prepare Tregs and a control line, CD45.2 2D2-FIG FOXP3^+^ Tregs were cultured in PC61 and IL-2 for either 13 or 40 days, and CD45.2 2D2-FIG conventional T cells (Tcons) were cultured in IL-2 for 13 days. To test the ability of Tregs to suppress naïve T cell activation, 25,000 CD45.2 2D2-FIG Tregs or 25,000 CD45.2 2D2-FIG Tcons were co-cultured with 150,000 CTV-stained CD45.1 2D2-FIG SPL responders for 5 days with 1 µM MOG35-55 and IL-2 (200 µl cRPMI). **(A)** CTV dye dilution and **(B)** percent suppression of CD45.1-gated responder T cells were analyzed by one-way ANOVA (*n* = 3). **(C–G)** A continuous line of FOXP3^+^ Tregs was activated with irradiated splenic APC, 1 µM MOG35-55, IL-2, and 100 pM TGF-β for 3 days. The PC61 monoclonal antibody was not added to these cultures to avoid adoptive transfer of antibody-coated T cells. Activated Tregs were cultured one additional day in IL-2 and were analyzed for expression of CD25 (clone 3C7) **(C)**, surface-bound PC61 (anti-rat IgG) **(D)**, and CD44 and CD62L **(E)**. Tregs were extensively washed and injected (3 × 10^6^/mouse) into naïve recipients on day −1. Recipients were then challenged with 100 µg MOG35-55 in CFA on day 0 and were given Pertussis toxin on days 0 and 2 to elicit experimental autoimmune encephalomyelitis (EAE). Significant differences (*p* < 0.05) were noted for the mean daily EAE scores on days 9–13 and 15–21 **(F)** and for daily weight loss on days 11–20. **(H,I)** EAE was elicited by administering 200 µg MOG35-55 in CFA on day 0 and Pertussis toxin on days 0 and 2. At peak disease (day 15, indicated by black arrow), activated 2D2 Tregs (3 × 10^6^/mouse) were administered i.v. by retro-orbital injection. Significant differences (*p* < 0.05) were noted for mean daily EAE scores on days 18–28 **(H)** and for daily weight loss on days 21–28 **(I)**.

Phenotypic analysis of activated Tregs revealed the activation-dependent upregulation of functional Treg markers including LAP, CTLA4, GARP, and TIGIT (Figure 9). These data reveal that TGF-β-conditioned activation of Tregs upregulates several markers associated with suppressive Treg function. To determine if continuously propagated FOXP3^+^ Tregs have suppressive capabilities, 2D2 Tregs were assayed for *in vitro* and *in vivo* suppressive activity. To assess *in vitro* suppressive activity, CD45.2 2D2 Tregs were cultured for either 40 or 13 days in IL-2 and PC61, whereas a control CD45.2 2D2 Tcon line was derived by propagation in IL-2 alone for 13 days. These Treg and control lines were then cultured with CTV-stained naïve responders from CD45.1 2D2 splenic leukocytes in the presence of MOG and IL-2 (Figure 10A). CTV dilution was measured in the CD45.1^+^ responders after 5 days as a measure of T cell proliferation. Suppressive activity was based on the percentages of hypo-proliferative CD45.1 2D2 T cells (Figure 10B). Responder 2D2 T cell proliferation was significantly inhibited by the presence of 2D2 Tregs when compared to cultures containing the 2D2 Tcon line. There was no difference in suppressive activities of Tregs rested for 40 or 13 days. These data provide evidence that Treg function is maintained when Tregs are continuously cultured with IL-2 and PC61.

A central question was whether these Tregs, representing the 2D2 MOG35-55-specific clonotype, exhibited suppressive activity in adoptive transfer experiments. To address this question, a continuous line of FOXP3^+^ Tregs was activated with irradiated splenic APC, 1 µM MOG35-55, IL-2, and 100 pM TGF-β for 3 days but without PC61 mAb to avoid coating the T cells with a rat IgG1 mAb that had depleting activity *in vivo*. Cell-surface PC61 remained high in the immediate aftermath of the activation culture, and thus the Tregs were cultured overnight in IL-2 to remove more cell-surface CD25/PC61 complexes. Phenotypic analysis of Tregs on the day of transfer showed that 93% were FOXP3^+^ CD25^high^ Tregs (Figure 10C). Most of the transferred Tregs had no or low detectable levels of PC61 on the cell surface as determined by staining with anti-rat IgG secondary antibody (Figure 10D). 65% of Tregs were CD44^high^CD62L^high^, whereas 33% of Tregs were CD44^high^ CD62L^low^ (Figure 10E). One day after adoptive transfer, mice were actively challenged to elicit EAE. The adoptive transfer of Tregs ameliorated severity of clinical paralysis and EAE-associated loss of body weight (Figures 10F,G). Recipients of Tregs (*n* = 6) differed from control mice (*n* = 5) in mean cumulative scores (12.2 ± 4.0 versus 26.8 ± 4.6; *p* = 0.0198), mean maximal scores (2.0 ± 0.7 versus 3.6 ± 0.2; *p* = 0.0343), mean maximal weight loss (12.5 ± 4.4 versus 28.7 ± 1.1%; *p* = 0.00475), and mean average weight loss (5.7 ± 3.3 versus 21.5 ± 2.4%; *p* = 0.00249). Mice that received Tregs had significantly lower mean daily clinical EAE scores on days 9–13 and 15–21 and significantly less weight loss on days 11–20 as assessed by the Student’s *t*-test. These data provided evidence that PC61 enables the selection of immunosuppressive FOXP3^+^ CD25^high^ Tregs that when appropriately activated have the ability to inhibit EAE in adoptive transfer experiments.

To address the Treg adoptive immunotherapy in a relevant preclinical model, adoptive transfer of activated FOXP3^+^ Tregs was performed in mice that were exhibiting severe paralytic EAE (Figures 10H,I). Mice were actively challenged with MOG35-55 in CFA to induce EAE on day 0 and then were separated into two groups that were matched for EAE severity and weight loss on day 15. Blastogenic FOXP3^+^ Tregs were previously prepared by activation for 3 days with irradiated bone marrow-derived dendritic cells, Con-A, MOG35-55, IL-2, Vitamin C, and TGF-β. These activation cultures were supplemented with Vitamin C due to the emerging awareness that the vitamin acts to promote functionality and stability to effector Tregs (32–36). Approximately 97% of cells expressed high levels of FOXP3, and these blastogenic Tregs had low to no detectable PC61. Adoptive transfer of these Tregs significantly alleviated severity of EAE based on a decrease in mean clinical score and an increase in body weight. When analyzed from days 20 to 28, mice that received Tregs (*n* = 4) differed from control mice (*n* = 5) in mean cumulative scores (13.3 ± 3.3 versus 28.4 ± 1.7; *p* = 0.003), mean maximal scores (2.1 ± 0.5 versus 3.8 ± 0.2; *p* = 0.0035), mean maximal weight loss (19.7 ± 0.9 versus 27.3 ± 2.2%; *p* = 0.0103), and mean average weight loss (12.7 ± 0.9 versus 22.8 ± 1.7%; *p* = 0.00102). The transfer of Tregs significantly alleviated the severity of EAE based on a decrease in mean daily clinical scores from days 18 to 28 and an increase in body weight from days 22 to 28. These data provided evidence that blastogenic 2D2 Tregs can mitigate EAE when administered in a therapeutic treatment regimen.

### Like the Bivalent PC61 mAb, a Monovalent PC61scFv Promoted the Dominant Outgrowth of FOXP3^+^ Tregs in Mixed Cultures

**Figure 11:**
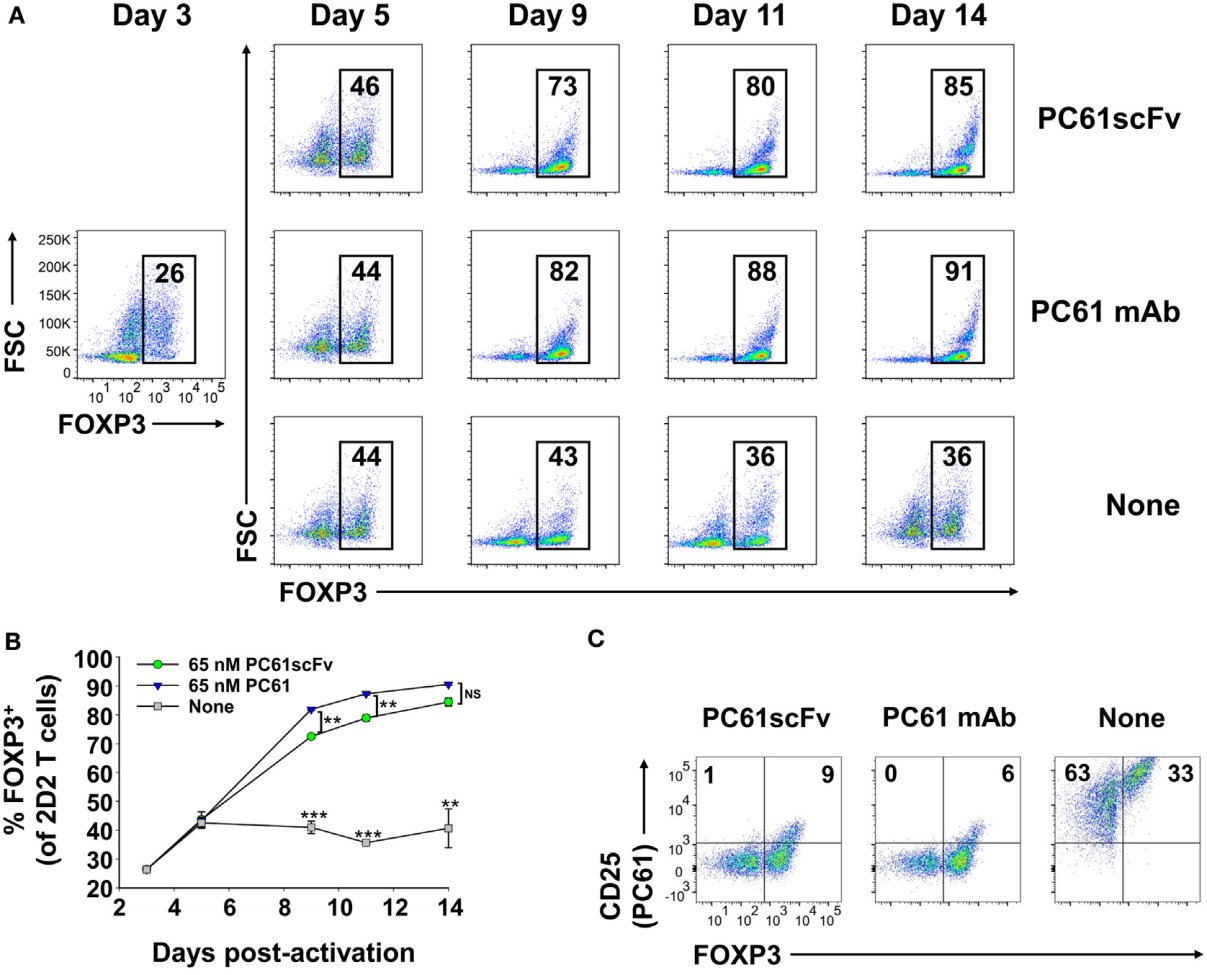
Like the intact PC61 monoclonal antibody (mAb), the monovalent PC61scFv stabilized regulatory T cell (Treg) lines. 2D2-FIG splenocytes were activated at a density of 2 × 10^6^ cells/ml in cRPMI with MOG35-55 (1 µM) in the presence of TGF-β (10 nM). After 3 days of activation, T cells were cultured (*n* = 2) at a density of 500,000 cells/ml in IL-2 together with 65 nM PC61scFv, 65 nM PC61 mAb, or vehicle. Cells were passaged under the same conditions every 3–4 days. **(A,B)** Representative dotplots and timecourse data show FOXP3^+^ Treg percentages in the Vβ11^+^ T cell population (values given at the top of each gate). ***p < 0.001, **p < 0.01, NS: not significant. **(C)** Vβ11^+^ cells were analyzed for expression of FOXP3 and the binding of an APC-conjugated PC61 mAb to surface CD25. These data are representative of three independent experiments.

We derived an expression system for a single-chain fragment variable (scFv) version of PC61 to assess whether a monovalent non-signaling version of PC61 retained Treg-stabilizing ability. This PC61scFv protein has the same binding specificity as PC61 (anti-CD25). However, unlike the intact PC61 mAb, PC61scFv was monovalent and therefore lacked cross-linking activity necessary for signaling. The PC61scFv also lacked constant region heavy chain domains and therefore lacked interactions with complement and FcγR. The PC61scFv is predicted to have qualitative advantages over intact PC61 in regard to tissue penetrance and absence of *in vivo* depleting activity. We tested the ability of this monomeric PC61scFv to stabilize Tregs in short-term cultures in comparison to the PC61 mAb (Figure 11). 2D2-FIG Tregs were activated with MOG35-55 in the presence of TGF-β for 3 days before being passaged into media containing IL-2 along with 65 nM PC61scFv, 65 nM PC61 mAb, or vehicle. Cells were passaged under the same conditions every 3–4 days. The presence of PC61scFv resulted in high Treg percentages (85% on day 14) indicating that PC61scFv mimicked the effect of PC61 mAb *in vitro* (Figures 11A,B). PC61scFv was slightly less efficient compared to the intact PC61 mAb, although this minor difference most likely was due to the lower affinity associated with monovalent binding, in contrast to the bivalent interactions of intact PC61 with CD25. The specificity of PC61scFv was confirmed by the ability of surface-bound PC61scFv to block the binding of a fluorochrome-conjugated PC61 mAb (Figure 11C). These data indicate that PC61 mediates Treg stabilization *via* CD25 neutralization rather than CD25 cross-linking.

## Discussion

Stable growth of autoreactive, TGF-β-induced FOXP3^+^ Tregs represents a critical prerequisite for effective Treg adoptive immunotherapies of autoimmunity and chronic inflammatory disease. However, the lack of viable strategies to maintain and expand functional CD25^high^ FOXP3^+^ Tregs in sustained culture has severely limited progress. Solutions to this problem represent a critical unmet need. The main issue is that FOXP3 expression in some Tregs wanes on a per cell basis over time, and otherwise stable FOXP3^+^ Tregs are overgrown by Tcons when cultured in IL-2, such that the cultured T cell population may acquire immune effector functions rather than suppressive regulation activity. This study provides an exquisitely simple solution to this problem. Addition of the anti-mouse CD25 antibody PC61 to a mixed culture of TGF-β-iTregs and Tcons in the presence of IL-2 led to the rapid establishment of cultures dominated by Tregs (>90% Tregs). This approach did not depend upon genetic modification or physical purification. These Treg lines indefinitely sustained a Treg lineage phenotype when maintained with low concentrations of IL-2 and high concentrations of PC61 in continuous culture. The Tregs derived in this study actively expanded and remained stable in the presence of PC61 and TGF-β in antigen-induced reactivation cultures, such that blastogenic Tregs expressed the prototypic Treg markers together with suppressive activity *in vitro* and *in vivo*.

### Elevated CD25 Expression on Mature Tregs Provides an Exploitable Treg Window

Previous studies showed that the potent IL-2 responsiveness of Tregs can be used to selectively promote Treg responses. For example, low-dose IL-2 therapies for type 1 diabetes selectively expanded existing Treg populations which could suppress islet cell destruction (8). Additionally, anti-IL-2 mAb/IL-2 immune complexes may target IL-2 to different T cell subsets depending on the epitope specificity of the anti-IL-2 mAb in the complex (14, 15, 37). The JES6-1 anti-IL-2 mAb/IL-2 immune complex appeared to target IL-2 to Tregs to favor Treg expansion. Conversely, the S4B6 anti-IL-2 mAb/IL-2 complex favorably expanded effector T cells by blocking the interaction between IL-2 and CD25. Advantages of targeting IL-2 versus CD25 have yet to be directly compared although targeting CD25, the Treg-specific component of the IL-2 receptor, may have qualitative advantages given the wide variations in endogenous IL-2 concentrations that may exist during chronic inflammatory autoimmune disease. Blockade of CD25 will predictably constrain IL-2 signaling across a broad IL-2 concentration range, with an upper threshold defined by those levels of IL-2 sufficient for CD25-independent IL2Rβγ signaling. That is, even with widely varying concentrations of IL-2 and cell surface CD25, mAb-mediated blockade of CD25 may provide a reliable clamp to ensure low-zone IL-2 signaling to promote dominant Treg responses.

The IL-2 concentration was instrumental in determining T cell subset dominance (Figures 6A,D). Low-intensity and high-intensity IL-2 signaling, respectively, supported Treg or Tcon subset dominance. The relation between low-zone IL-2 signaling and Treg responses reflected superior CD25 expression and exquisite IL-2 sensitivity of Tregs. The relation between high-zone IL-2 signaling and Tcon subset dominance may reflect differences in the respective IL-2 signaling pathways. IL-2 signaling in Tregs is mediated primarily through the JAK/STAT pathway whereas IL-2 signaling in Tcon subsets is mediated robustly through both JAK/STAT and PI(3)K pathways. PTEN, the PI(3)K inhibitor, is selectively expressed in Tregs and is important in stabilizing the Treg phenotype (38), whereas PTEN is downregulated in Tcon subsets (39). Thus, IL-2 has superior potency for Tregs due to higher CD25 expression, which confers Treg dominance at low IL-2 concentrations. By contrast, IL-2 has superior efficacy for Tcons due to robust signaling through both JAK/STAT and PI(3)K pathways, which confers Tcon dominance at high IL-2 concentrations. Thus, differential expression of CD25 and PI(3)K signaling pathways provide qualitative distinctions in IL-2 signaling pathways that are foundational for the specialized Treg and Tcon niches.

Chronic CD25 blockade was the key to exploitation of the Treg window and stabilization of FOXP3^+^ Tregs. However, anti-CD25 mAbs varied substantially in Treg stabilization activity. The mAb of choice for stabilization of mouse Tregs was PC61, which was a stronger inhibitor of IL-2-dependent proliferation than the anti-CD25 mAbs 3C7 and 7D4 (Figure 5A). The use of PC61 in concert with low IL-2 concentrations provided the appropriate qualitative and quantitative signals necessary for stabilization of FOXP3 expression and maintenance of the Treg phenotype. Although PC61 was not useful in initial activation of 2D2-FIG SPL with antigen and TGF-β, PC61 in concert with IL-2 was included in all subsequent cultures including maintenance cultures and reactivation cultures. TGF-β was beneficial during the initial activation culture and in all subsequent reactivation cultures. Thus, PC61 worked in concert with IL-2 to maintain Tregs, and TGF-β worked in concert with MHCII-restricted antigen to stabilize Tregs during blastogenic expansion. PC61 was the technical tool that allowed exploitation of a Treg window, which was operationally defined by a low range of IL-2 signaling that enabled dominant outgrowth of stable FOXP3^+^ Tregs. If IL-2 concentrations in PC61-supplemented cultures exceeded this range, then PC61 lacked Treg stabilization activity, and Tcon subsets dominated the culture.

An important consideration was whether PC61 mAb crosslinked CD25 to elicit qualitatively unique, non-canonical IL-2 signaling pathways to stabilize Tregs. CD25 has no known intracellular signaling activity, and IL-2 signaling is mediated through the cytoplasmic domains of CD122 and CD132 (40). Nonetheless, CD25 has a cytoplasmic domain, and CD25 cross-linking may indirectly crosslink other associated proteins. Hence, our studies with intact PC61 mAb could not exclude the possibility that PC61 may qualitatively modulate IL-2 signaling to favor the Treg subset. To address this question, we derived a monomeric PC61scFv that lacked cross-linking activity. This monomeric PC61scFv had essentially the same activity as PC61 mAb in Treg stabilization assays (Figure 11). These results support the conclusion that PC61 stabilizes Tregs primarily through preferential blockade of IL-2 signaling in Tcon subsets. Notably, the use of PC61 or PC61scFv was fully sufficient for derivation of long-term stable FOXP3^+^ Treg lines without any physical purification or genetic modification. The use of suitable anti-CD25 reagents could be used to guide formulation of culture conditions suitable for derivation of enriched Treg populations in large culture settings or bioreactors.

### Low-Zone IL-2 Signaling Confers Treg Stability

The IL-2 pathway is critical for self-tolerance in that genetic deficiencies in the major components of the IL-2/CD25/IL2Rβγ/STAT5/JAK3 pathway result in systemic autoimmunity (41–46). The paradox is that IL-2 is the prototypic T cell growth factor *in vitro* that drives dominant outgrowth of CD4^+^ and CD8^+^ effector lineages. Several studies showed that IL-2 is required for induction and maintenance of FOXP3 expression in Tregs (47), but IL-2 also drives proliferation of Tcons and destabilization of Treg populations (Figures 2 and 6). This study provides insight into the “IL-2 paradox” by showing that continuous culture of Tregs in IL-2 and PC61 resulted in maintenance of high levels of FOXP3 expression and Treg stability for more than 60 days without any phenotypic or functional attenuation (Figure 7). Indeed, continuous culture in IL-2 and PC61 appeared to promote a canonical Treg phenotype as measured by progressive increases in the expression of Helios and Neuropilin-1 (Figure 7). These findings provide suggestive evidence that the duration of a Treg phenotype might be associated with progressive stabilization of a FOXP3^+^ Treg phenotype, such that Tregs may gravitate to a more differentiated phenotype with time. If continuous low-zone IL-2 signaling in the context of a Treg window is key for understanding stabilization of FOXP3^+^ Tregs *in vitro*, the implication is that continuous low-zone IL-2 signaling may also be key for maintenance and progressive differentiation of Tregs *in vivo*.

TGF-β was also required for Treg stability, but only in the context of MHCII-restricted antigen presentation by splenic APC in activation-dependent expansion cultures. TGF-β had no effect in maintenance cultures when added during propagation in IL-2 with or without PC61. In reactivation cultures, a TGF-β-blocking antibody led to accelerated Treg destabilization, whereas addition of exogenous TGF-β preserved FOXP3 expression and FOXP3^+^ Treg percentages at optimal levels (Figure 8). In reactivation cultures that included IL-2 and TGF-β, Tregs expanded more than fivefold without loss of FOXP3 as both a percentage of the T cell population and on a per cell basis based on MFI. The concentration of TGF-β in the reactivation culture was tailored to the TCR strength for cognate antigen of the particular clonotype. In other words, the correct balance of TGF-β, antigen, and IL-2 was necessary for optimal expansion of Tregs and maintenance of the Treg phenotype. For example, 100 pM TGF-β was sufficient for expansion of stable MOG-specific Tregs, which represents a low affinity clonotype. Higher concentrations of TGF-β (1 nM) were needed for optimal expansion of stable OVA-specific Tregs, which exhibits much stronger antigenic potency. Thus, TGF-β was needed in sufficient concentrations to counter-balance antigenic strength but these TGF-β concentrations had to be below thresholds that completely blocked the antigenic response. Thus, like the Treg window established for PC61/IL-2 in maintenance cultures, an appropriate range of TGF-β concentrations was needed for optimal stabilization of Tregs during antigen-reactivated expansion cultures.

For both IL-2 and PC61 in maintenance cultures and TGF-β in reactivation cultures, a major question was whether Treg stabilization reflected the intrinsic stabilization of FOXP3 expression or whether differential growth (or death) rates in Treg and Tcon subsets determined final Treg percentages. The most likely scenario is that both mechanisms contributed in part to the derivation of continuous Treg lines. For example, Tregs cultured in the presence of PC61 and IL-2 had higher FOXP3 expression on a per cell basis than Tregs cultured in IL-2 alone, and Tregs cultured in PC61 and IL-2 also had higher growth rates than the Tcon subsets. PC61 may suppress Tcon proliferation which in turn may suppress secretion of pro-inflammatory cytokines that could antagonize TGF-β and destabilize FOXP3. These considerations may reflect a fundamental competition between these T cell subsets, wherein Treg subsets inhibit Tcon subsets, and Tcon subsets inhibit Treg subsets *via* mechanisms of reciprocal inhibition.

An important caveat is that this study pertains to the MOG-specific 2D2 clonotype, the OVA-specific OTII clonotype, and mitogen-activated polyclonal C57BL/6 T cells. The kinetics by which PC61 selects for CD25^high^ Tregs will be determined in part by the relative proportion and persistence of CD25^high^ Treg and Tcon subsets in the cell preparation, which may vary in different T cell preparations. Although PC61/IL-2 selection exploits qualitative distinctions in CD25 expression in Treg and Tcon subsets, stronger antigenic or mitogenic stimuli may cause more persistent CD25 expression in Tcon subsets which may necessitate longer periods of PC61/IL-2 selection to achieve FOXP3^+^ Treg dominance. PC61-mediated Treg selection therefore may vary in kinetics based on the inherent antigenic reactivity of the relevant clonotype. A second caveat is that PC61/IL-2 selects CD25^high^ FOXP3^+^ subsets at the expense of CD25^low^ FOXP3^+^ subsets. For example, pre-existing Tregs isolated from secondary lymphoid organs express CD25 across a quantitative continuum, and PC61/IL-2 selects the CD25^high^ subset that represents the functionally mature suppressive subset.

## Conclusion

This study defined an operative “Treg niche” defined by low-zone IL-2 signaling. This study also provided a strategy for maintenance and expansion of stable FOXP3^+^ Treg lines *in vitro*, which could be propagated indefinitely without phenotypic instability. These principles were validated for TGF-β-inducible Tregs, which may be optimal for derivation of antigen-specific Tregs from peripheral blood. Although we assume that these principles would also apply to thymic or natural tTreg/nTregs, this issue awaits future study. Overall, this strategy could be applied broadly to Tregs of any clonotypic specificity and thereby may have applicability for the advancement of antigen-specific Treg-based immunotherapies.

## Ethics Statement

Animal care and use was performed in accordance with approved animal use protocols and guidelines of the East Carolina University Institutional Animal Care and Use Committee.

## Author Contributions

DW and MM designed the project and wrote the manuscript. DW performed the majority of the experiments, and DG, RN, CM, and MM contributed to the experimentation. All authors analyzed the data, provided intellectual input, and edited the manuscript.

## Conflict of Interest Statement

The authors declare that the research was conducted in the absence of any commercial or financial relationships that could be construed as a potential conflict of interest.
